# Slip deficit and temporal clustering along the Dead Sea fault from paleoseismological investigations

**DOI:** 10.1038/s41598-018-22627-9

**Published:** 2018-03-14

**Authors:** Marthe Lefevre, Yann Klinger, Mahmoud Al-Qaryouti, Maryline Le Béon, Khaled Moumani

**Affiliations:** 1 Institut de Physique du Globe de Paris, Sorbonne Paris Cité, UMR 7154 CNRS, F-75005 Paris, France; 2 Jordan Seismological Observatory, Ministry of Energy and Mineral Resources, Amman, Jordan; 3 Department of Earth Sciences, National Central University, Taoyuan, Taiwan; 4 Geological Mapping Division, Ministry of Energy and Mineral Resources, Amman, Jordan

## Abstract

Temporal distribution of earthquakes is key to seismic hazard assessment. However, for most fault systems shortness of large earthquake catalogues makes this assessment difficult. Its unique long earthquake record makes the Dead Sea fault (DSF) exceptional to test earthquake behaviour models. A paleoseismological trench along the southern section of the DSF, revealed twelve surface-rupturing earthquakes during the last 8000 years, of which many correlate with past earthquakes reported in historical chronicles. These data allowed us building a rupture scenario for this area, which includes timing and rupture length for all significant earthquakes during the last two millenaries. Extending this rupture scenario to the entire DSF south of Lebanon, we were able to confirm the temporal-clustering hypothesis. Using rupture length and scaling laws, we have estimated average co-seismic slip for each past earthquake. The cumulated slip was then balanced with long-term tectonic loading to estimate the slip deficit for this part of DSF over the last 1600 years. The seismic-slip budget shows that the slip deficit is similarly high along the fault with a minimum of 2 meters, which suggests that an earthquake cluster might happen over the entire region in the near future.

## Introduction

Successions of intense periods of seismic activity rupturing significant length of a fault followed by longer periods of seismic quiescence have been documented along several strike-slip faults^1–6^, suggesting that temporal clustering of earthquakes might be a common behaviour for major strike-slip faults. Testing this assumption, however, has long been hampered by the lack of consistent earthquake time series for long-enough fault sections. Moreover, temporal clustering remains a critical issue for seismic hazard models that only started to be addressed in the most recent modelling attempts.

**Figure 1:**
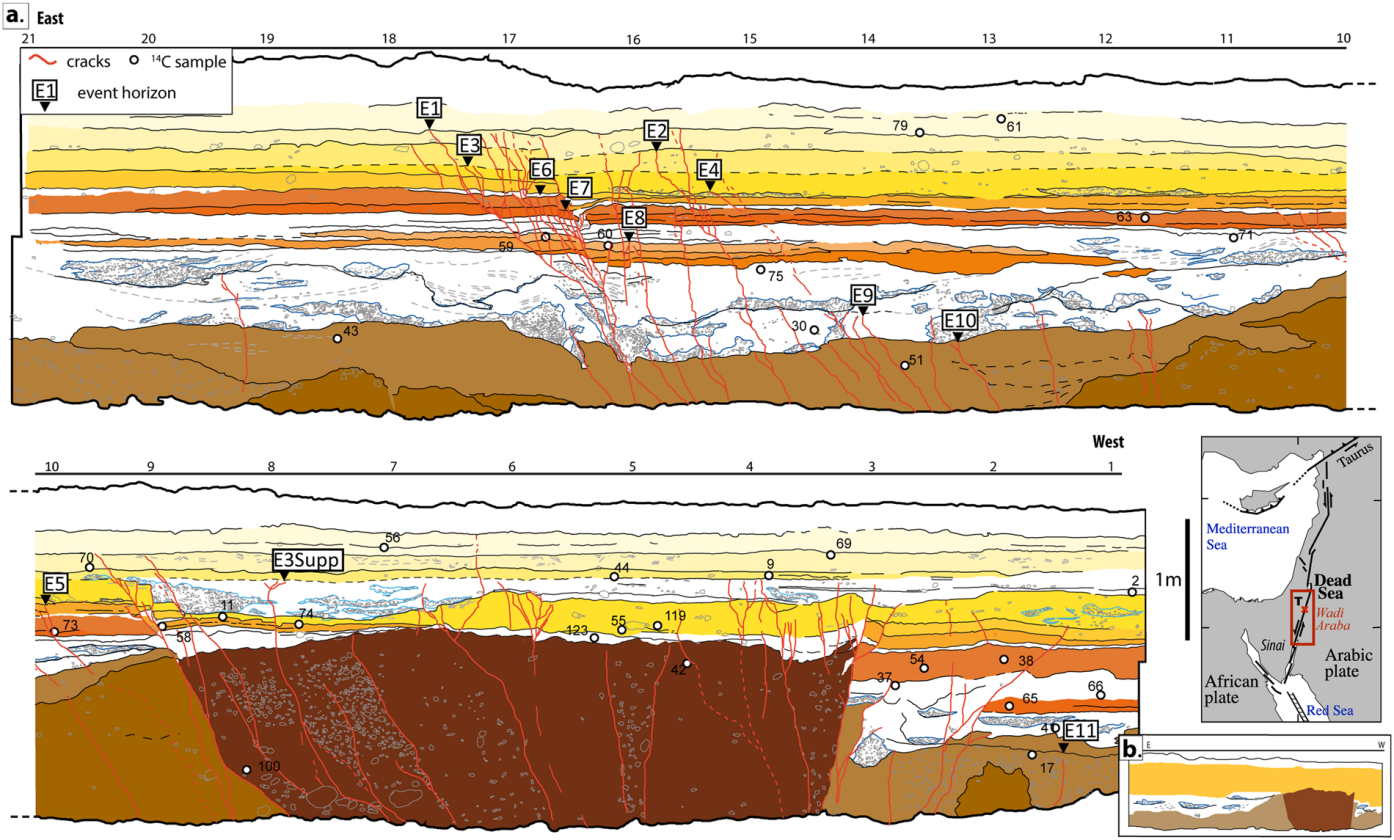
(**a**) Detailed log of the southern wall of the trench. Units are differentiated according to facies. White dots indicate locations of 14 C samples collected from both walls (the samples collected from the northern wall are represented at a stratigraphically and structurally equivalent location on the southern wall log). (**b**) Simplified stratigraphic section of the trench, only the four main units are represented, in dark brown the unstratified unit associated to push-up, in light brown the coarse alluvial unit, in white the channelized unit, and in yellow the succession of flat sandy layers. **Inset map** shows the Levantine area with the entire Dead Sea fault, tectonic features are derived from Garfunkel *et al*.^54^, the red square highlights the Wadi Araba and correspond to the location of Fig. 2, the site of Taybeh is marked (T). Figure was generated with Adobe illustrator CS6 (http://www.adobe.com/fr/products/illustrator.html).

The DSF, a 1200 km-long continental strike-slip fault, is the tectonic boundary between the Arabian plate and the Sinai micro-plate in the eastern Mediterranean region^7,8^ (Fig. 1). Previous works, based on geological data and reassessment of historical records, have hinted at seismic temporal clustering along some sections of the Dead Sea fault^6,9,10^. Recently, the occurrence of the Mw 7.3 earthquake in the Gulf of Aqaba in 1995, the only large event along the entire Dead Sea fault for more than 200 years^11,12^, revived the question of the possible onset of a new earthquake series during the upcoming decades.

The DSF area has long been inhabited and it provides a unique historical archive including numerous earthquake testimonies^13–17^. In several places these archives have been complemented by paleoseismological investigations to better locate past earthquakes and to expand earthquake catalogues in time^18–24^. However, the 180 km-long southernmost section of the DSF on shore, named the Wadi Araba fault, remains less well known due to a lower population density. Hence, we opened a trench in the Wadi Araba to close the gap of paleoseismological data (Fig. 1). These new data were then integrated with the corpus of data already available to propose an earthquake catalogue including timing and rupture length for significant earthquakes for at least the last 1600 years, for the area from southern Lebanon to the Gulf of Aqaba.

This new dataset allowed us, using seismological scaling laws^25^, to estimate average co-seismic deformation accommodated by earthquakes during the last 1600 years. It was then compared to regional tectonic strain accumulation, in order to assess the current slip deficit along the different fault sections. Finally, to better evaluate the seismic hazard associated with this fault, which lies close to several major cities such as Jerusalem, Amman or Damascus, we tentatively computed probabilities of occurrence of M > 7 earthquakes for different time periods, using simplified assumptions about earthquake recurrence.

## Paleoseismological Observations

**Figure 2:**
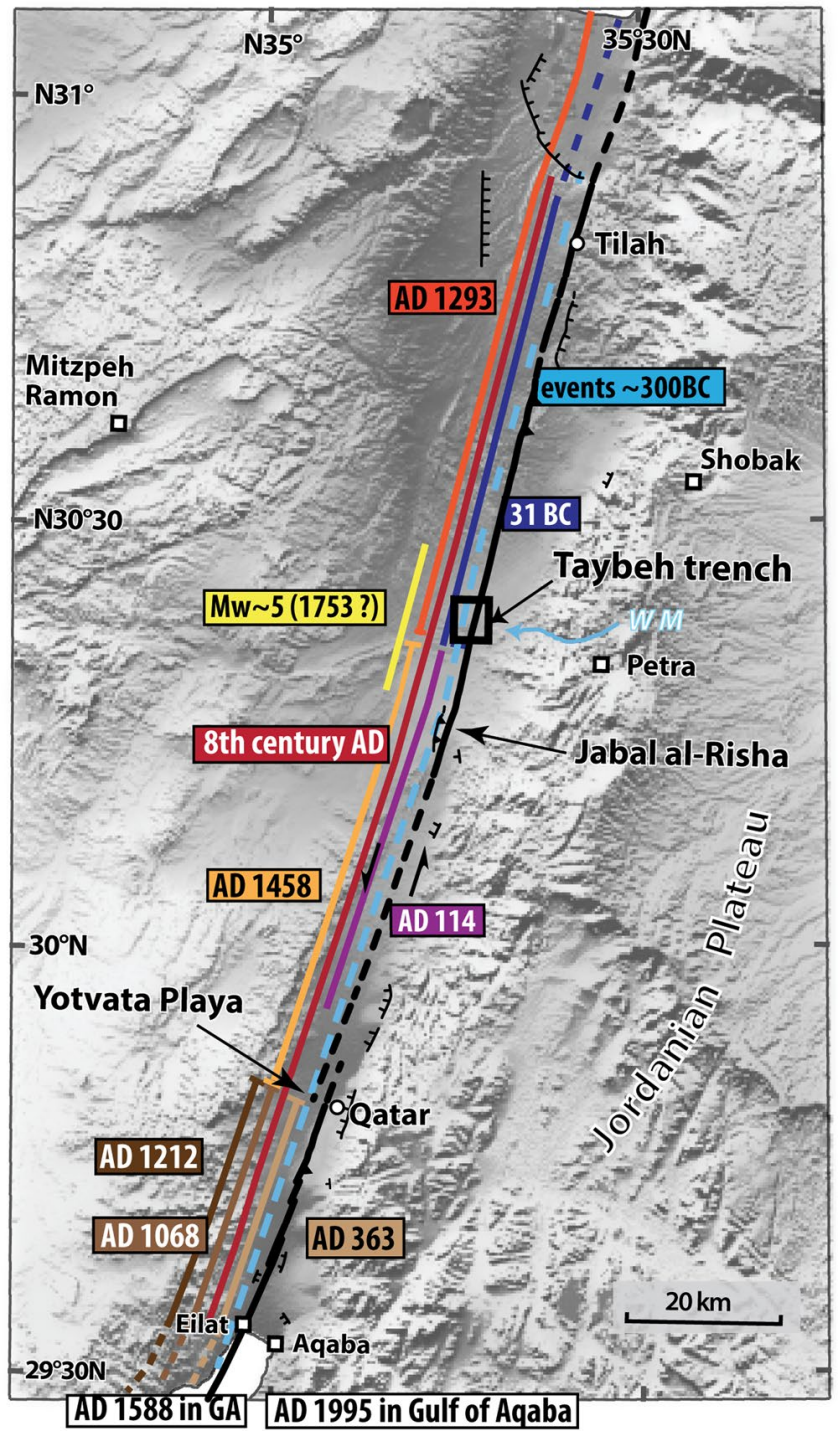
Topographic map of the Wadi Araba from SRTM3 (pixel size, ~90 m) with lateral extent of historical earthquakes based on events identified in trenches and in historical accounts. Locations of the 1068 AD, 1212 AD, 363 AD events not identified in the Taybeh trench are from Klinger *et al*.^24^. The Wadi Araba fault segments were reproduce after Le Béon *et al*.^46^. The Jabal al-Risha compressional jog and the Yotvata extensional jog appear to act like stopping/initiation points for many ruptures, although larger earthquakes seem to be able to break through. WM: Wadi Musa. Figure was generated with Adobe illustrator CS6 (http://www.adobe.com/fr/products/illustrator.html).

The Wadi Araba fault section is mostly linear with two noticeable fault jogs, the Yotvata playa, which is an extensional relay zone, and the compressional bend located at the Jabal al-Risha. They are located respectively about 30 km and 100 km north of the city of Aqaba (Fig. 2). At Jabal al-Risha, the fault strike changes from N17E southward to N12E northward over 20 km, producing a low relief that locally blocks westward-flowing drainages^26^.

To test how this compressional bend might impact earthquake propagation along the Wadi Araba fault, we dug a trench 20 m long and 2.5 m deep at a site called Taybeh (30°22′40.71″N, 35°16′30.84″E). At that site, the topography associated with the compressional bend traps fine sediments while diverting the Wadi Musa, which is flowing westward from the Jordanian plateau (Fig. S1). The main active strike-slip fault runs across the distal part of the Wadi Musa alluvial fan where the fault location is only indicated by small push-ups popping up through the surface of the fan.

Three trench walls were excavated (one wall facing south, and two peels facing north) that are summarized hereafter (full description of stratigraphy and evidence for individual earthquake identification are provided as electronic sup.). The stratigraphy exposed in the trench is quite diverse. Four main units can be distinguished (Figs 1a,b; S2 and S3). The lower unit consists of coarse alluvial deposits. Nested channels including gravels to fine sand characterize the middle unit. The upper section of the trench is composed of a 1 m-thick succession of distinct laminated sandy layers. Lastly, a massive reddish sandy unit is popping up through the trench stratigraphy between marks 3 and 9, which is interpreted as a small push-up, based on strong similarity in material and appearance with the observed push-up located a hundred meters south of our trench (Fig. S1). Moreover, this unit is affected by numerous fractures defining a positive flower structures consistent with compressional deformation. Stratigraphic relations suggest that the top part of the push-up was eroded with subsequent deposition of sediments on top of it. At marks 9 and 7, some of these younger sediments show signs of apparent normal faulting that necessarily post-date the emplacement of the push-up, emphasizing the changing nature of small-scale vertical deformations along major strike-slip ruptures during successive earthquakes.

Although, deformation is visible throughout most of the trench, it is more pronounced around mark 17, where deformation is mostly characterized by normal displacement and cracks, while evidence of strike-slip motion remains elusive.

We identified event horizons based on offset layers and consistent groups of cracks (a minimum of 4 different cracks in that case) ending at the same stratigraphic level^27^. However, the central push-up disrupts the general stratigraphy, making it difficult to unambiguously establish the lateral continuity of these event horizons between the eastern and the western part of the trench. Therefore we also had to rely on ^14^C dating of charcoals to strengthen the stratigraphic sequence across the trench. Thirty-two charcoals distributed over the three trench walls were dated by accelerator mass spectrometry (Tablesup1; Fig. S4). The chronological sequence was then refined using a-priori information derived from stratigraphic relationship of ^14^C samples, following a Bayesian analysis approach in Oxcal^28^.

**Figure 3:**
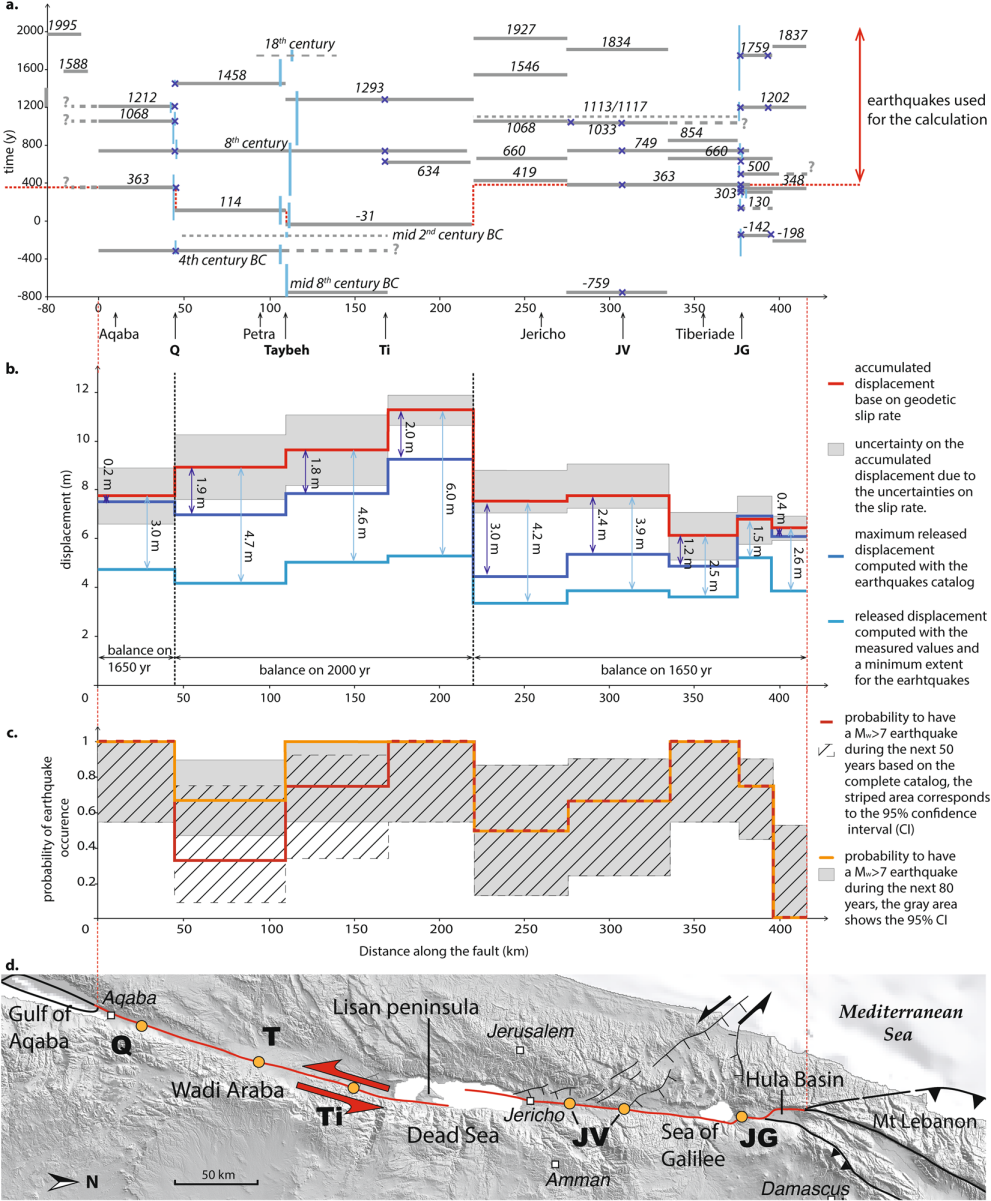
(**a**) Historical earthquakes distribution in space and time, each horizontal bar corresponds to an approximate earthquake location along the Dead Sea fault, dashed lines are used for uncertain locations or lateral limits. The vertical bars correspond to the time interval associated to the paleoearthquakes at Taybeh, obtained after a Bayesian modeling in Oxcal. For Qatar the time intervals are from Klinger *et al*.^24^, for the Jordan Gorge the ages are from Marco *et al*.^5^ and from Wechsler *et al*.^40^. The blue crosses represent the earthquakes visible in the different trenches. (**b**) Accumulated displacement (red) from tectonic loading and cumulated released slip (blue) due to major earthquakes from 350AD to 2015AD along DSF, between the Gulf of Aqaba and the Hula basin. For all earthquakes two scenarios are considered, the dark blue line shows the released displacement linked to a maximum lateral extent, the light blue line shows the released displacement linked to a minimum lateral extent. The slip deficit is written in meter for all the sections and for the two scenarios. (**c**) Probability of M ≥ 6.5/7 earthquakes for the next 50 and 80 years (respectively red and orange) calculated with the ‘empirical’ method of Savage^52^. (**d**) Simplified structural map of the southern Dead Sea fault, the studied branches are highlighted in red, the sites of previous paleoseismological studies in the area are shown, Q: Qatar, Ti: Tilah, JV: Jordan valley. The topography is from SRTM3 and the faults geometries are derived from Le Béon *et al*.^26^. Figure was generated with Adobe illustrator CS6 (http://www.adobe.com/fr/products/illustrator.html).

Eventually, the Taybeh trench displays a 8000-year-long sedimentological record, without major sedimentary hiatus. The dates of the seismic events span from the 7^th^ millenary BC to the 18^th^ century AD (Figs 3a; S5; Tablesup 1). Detailed description about specific association between earthquakes observed in the trench and historical events is found in the supplementary materials, and here after correlations are only summarized. Among the 12 identified events, the oldest three cannot be associated with specific historical earthquakes due to loosely-constrained ages and scarce to nonexistent testimonies. However, we correlate the next 8 earthquakes with historical events: The correlation for events E8 to E6 remains relatively uncertain due to the lack of historical reports, nevertheless we suggest that event E8 is associated with the mid 8^th^ century BC event, event E7 with the mid 4^th^ century BC event and event E6 as the mid 2^nd^ century BC event. From event E5, association between our observations and historical catalogues is stronger and only these events will be used in the following part of this work: E5 is associated with the 31 BC event and E4 with the 114 AD event. We correlate E3 with the 8^th^ century AD crisis, E3bis with the 1293 AD event and E2 with the 1458 AD event (Fig. S5). The most recent event is dated around the 18^th^ century AD, a time when no major earthquake is documented in the area. Hence, we assume that it is a smaller-magnitude earthquake, such as Mw5 to Mw6 event. Indeed, Liu-Zeng *et al*.^29^ showed that under favorable conditions surface rupture of local moderate-magnitude earthquakes could be preserved in the stratigraphy.

## Earthquake rupture length in the Wadi Araba

Trenches provide local information on ground-rupturing earthquakes. Hence, combining several trenches helps determining the rupture length of past events, which is a proxy for the earthquake magnitude^25^. To establish the rupture length of past earthquakes for the Wadi Araba fault, we combined direct evidence of ruptures at our new site of Taybeh, with evidence from the site of Qatar^24^, about 30 km north of the city of Aqaba, and from the site of Tilah^21^, located just south of the Dead Sea basin (Fig. 2). These data were complemented by information regarding historical destructions^16^, including in Aqaba and Petra^30^ and by observation of seismites in the Dead Sea basin^31^. Moreover, as earthquake ruptures tend to initiate or end at major jogs^32,33^, at first order fault segmentation based on such geometrical asperities provided a template for potential ruptures scenarios.

In the following, we detail observations and our assessment of rupture length for three events, 31 BC, 114 AD and 1458 AD, characteristic of the different cases we encountered. A full description of the same process for all events considered in our study can be found in supplementary materials.

Two events are observed only in the Taybeh trench and are hardly reported in local historical chronicles, the 31 BC and the 114 AD events, making determination of rupture length difficult. The 31 BC event is documented in our trench and recognized at several places in seismites of the Dead Sea, with larger deformation in the southern part of the basin^31^. Damage was reported in the area around Jerusalem, although the severity of the damage remains arguable^16^. Hence, we favored a rupture of the northern segment of the Wadi Araba fault ending in the Dead Sea. The 114 AD event is even less documented. Some damage is reported in Petra and along the road from Petra to the Mediterranean sea^16^ that are possibly associated with an earthquake. The timing of a limited group of cracks in the Taybeh trench is also consistent with these reports, which would confirm the occurrence of an earthquake at that time. However the scarcity of evidence indicates that, if it did occur, it was probably a moderate magnitude event. Hence, we assume that it ruptured partially the central segment of the Wadi Araba fault, as it is not found at the Qatar site^24^.

For better documented events, we firstly consider the sites where a surface rupture associated with the event is visible to determine a minimum lateral extent of the rupture. We then use the historical data to refine the limits of the rupture. For example the 1458 AD earthquake is recognized both in Taybeh and in Qatar^24^, indicating that the rupture was at least 65 km long. It did not seem to have extended further southward as no damage was reported in Aqaba. To the north, an event with an overlapping age is found in the seismites of the Dead Sea^31^ and damage is documented in several places in the south of the Dead Sea. However, this event seems to be absent from the paleoseismological record at the site of Tilah^21^, suggesting that it did not rupture up to the Dead Sea and was limited to the central part of Wadi Araba.

Eventually, combining the new constrains brought by the Taybeh trench with data already available, we refined the regional historical catalogue of seismicity, possibly confirming the existence of the 114 AD earthquake, and we estimated rupture length for most past earthquakes along the Wadi Araba fault during the last 2000 yrs (Fig. 2).

## Slip deficit assessment and implication for seismic hazard

To test the hypothesis of temporal clustering along the southern DSF, we expanded the earthquake catalogue for the area from southern Lebanon to the Gulf of Aqaba based on published literature (literature review in supplementary mat.) (Fig. 3a). As for the Wadi Araba fault, based on location of major jogs and bends we defined nine segments along the southern DSF, which are from south to north: three segments in the Wadi Araba, the Lisan peninsula segment, the Jericho segment, two segments in the Jordan valley, the Jordan Gorge segment and the Hula basin segment (Fig. 3d).

Then, historical earthquakes were systematically associated with one or several fault segments in a pattern that ensures consistency with available paleoseismological data^21–24,34,35^ and archaeological data^13,36,37^, as it was done for the Wadi Araba. Figure 3a summarizes the distribution of Mw >6.5/7 earthquakes on the Dead Sea fault, including rupture length. It also includes a few lower-magnitude events, such as the 1834 event, which are poorly described despite being quite recent. The proposed catalogue does not cover the same time period for all fault sections, because different paleoseismological records do not span the same time period. Moreover, historical records are uneven and usually scarcer in less densely populated areas.

Overall, our catalogue shows that the seismic activity of the DSF is not regular through time. Short periods, lasting one to two centuries, during which the entire fault ruptures in a succession of earthquakes, alternate with longer periods of roughly 350–400 years with very limited seismic activity. Two periods of such intense activity can be recognized during the 8^th^ and the 12^th^–13^th^ centuries AD. A third period might also be identified during the 4^th^ century, although ruptures along the Wadi Araba are missing, either because they were not recognized or that they did not happened (Fig. 3a). During each earthquake cluster the entire fault eventually ruptures, although the spatial distribution of earthquakes seems to be random and does not follow any obvious neighboring-segment triggering pattern. Limited data availability makes it difficult to assess the extension of a similar pattern of temporal clustering further north, in Lebanon and Syria. However, historical and paleoseismological data suggest that the period of the 12^th^ to 13^th^ centuries AD, at least, was also a period of intense seismic activity along the northern section of the DSF^19,38,39^, pointing out that temporal clustering might dominate the seismic activity along the entire DSF.

Using the assumed rupture length of each earthquake presented in the catalogue, we can estimate the average coseismic displacement accommodated by these earthquakes. We compared this displacement with the accumulated slip due to tectonic loading. Since the seismic activity seems clustered, we needed to consider several seismic cycles to average out slip variability. Hence, we included the longest possible time period where we consider we have a complete catalogue of significant earthquakes for the entire fault, starting back in the 4^th^ century AD. This time window comprises at least 3 seismic crises, *i.e*. several significant earthquakes for each fault section, and it should be representative of the fault behaviour, including short-term variability. We assumed, as an initial condition, that just after the seismic crisis of the 4^th^ century AD, most of the stress had been released everywhere along the fault. For the three segments that did not rupture during the 4^th^ century AD crisis, the Lisan peninsula and northern parts of Wadi Araba, we extended the time period to the closest earthquakes before the 4^th^ century AD, respectively in 31 BC and 114 AD.

The total co-seismic displacement was computed by summing up average displacement associated to each earthquake. The latter was obtained by combining the hypothesized rupture length for each earthquake in the catalogue with the empirical law relating the surface rupture length (L) to the average displacement (AD) for strike-slip earthquakes: log(AD) =−1.7 + 1.04 log(L)^25^. In the computation we considered two extreme scenarios, corresponding respectively to the maximum and minimum released co-seismic displacement. In the first one, for each earthquake, we use the maximal value for the rupture length that would still be consistent with all the observations. For example, for the 8^th^ century seismic crisis, in the Wadi Araba we considered one single rupture, about 210 km-long, rupturing the entire Wadi Araba. Conversely, in the second scenario we assigned the shortest possible rupture length to each earthquake. Therefore, in the case of the 8^th^ century crisis we considered a series of three smaller-magnitude events, one on each segment (60 km long in average). In addition, for the second scenario, specifically for the Jordan Gorge section, we used direct measurements of earthquake displacements instead of modeled values, based on 3D paleoseismic observation^40^. These displacements are smaller than values derived from scaling laws^25^. Part of this difference might be explained by the trench location at the end of the fault section. Direct offset observations are also available in the Wadi Araba^21,41^, at Tilah an aquaduc is offset by 1.6 ± 0.4 m^21^ and a water reservoir by 2.2 ± 0.5 m^41,42^. These values are consistent with values predicted by scaling laws^25^. When available, we include earthquake magnitude. For our area, only the magnitude Mw ~6.3 of the 1927 AD event was recorded^43^. The magnitude Mw ~6.3 is smaller than the magnitude that would be derived from the scaling laws using the rupture length associated to the 1927 event. Hence, in our lower slip scenario we use a slip value consistent with the Mw ~6.3 magnitude, rather than slip directly modeled from rupture length. Eventually, despite the length of the host faults, we considered for the computation that the 1068 AD, 1113/1117 AD event and 1834 AD event were of smaller magnitude, around 6.5–6.9, because the historical reports for these events are vague and suggest limited damage. In general taking into account measured values of displacement participates to lower the stress released per event.

We acknowledge that the segmentation we use and the fact that all events rupturing the same section of fault have the same rupture length are oversimplification. However, a sensitivity test shows that for our average fault-section length of 60 km, a variation of 20% of the rupture length would lead to an average change in slip of about 0.24 m, which is not very significant when considering the total cumulative slip. Indeed, because interpretation of historical data in some cases remains arguable, alternative scenarios for lateral extent of historical earthquakes, that would affect the distribution of cumulative slip to some extent, could never be totally ruled out (see alternative scenarios in sup. mat.). We favore here a scenario that is consistent with all available data and that minimizes assumptions.

In parallel, we computed the accumulated strain due to tectonic loading over the same time period for each fault section. Comparison of geologic and geodetic slip rates along the DSF shows that the slip rate is steady over the Holocene^44–46^. Hence, for our calculation we used the geodetic rates published for the different fault sections, which are consistent in the limit of uncertainties all along the fault: For the Wadi Araba and the Lisan peninsula segments we used respectively a slip rate of 4.7 ± 0.7 mm/yr, and 5.5 ± 0.3 mm/yr^47^, for the Jordan valley we used a rate of 4.7 ± 0.5 mm/yr^48^, and for the Jordan Gorge and the Hula basin segments we used a rate of 4.1 ± 0.8 mm/yr^49^. On the northern segment of the Jordan valley Hamiel *et al*.^49^ suggest that 10% of the seismic moment is released by a 1.5 km-thick surface creeping layer, which contributes to reduce the accumulated constraint. For this segment we calculate an equivalent slip rate of 3.7 mm/yr, which includes the reduction of accumulated seismic moment.

When we compare the cumulative deformation accommodated by earthquakes with the tectonic strain accumulated over the same period, we found that there is a significant deficit of seismically released strain for most fault sections, even when one considers the scenario with the maximum rupture length for each event, *i.e*. the largest magnitude and slip per event (Fig. 3b). In this maximum scenario, on average about 2 meters of slip deficit accumulated during the last 1600 years all along the fault, which would correspond to a magnitude Mw 7.3 event on each section to fully release the current accumulated slip. However, the amount of slip to be released might be lower in this area, if one considers the extremely low level of current microseismicity in the Wadi Araba^50^ as the signature of very large earthquakes rupturing deeper than classical seismogenic crust and associated with very large slip^51^. The alternative scenario, involving smaller-magnitude earthquakes, implies a more significant slip deficit, around 5 m for most of the fault sections (Fig. 3b), which should translate either in a flurry of magnitude 7+ events or in much larger magnitude events (>7.5), which have never been reported yet south of Lebanon.

In both scenarios, even if the seismic history of each section differs, it is worth notice that the difference between the accumulated and the released slip appears to be relatively uniform along the entire fault, which argues in favor of earthquake temporal clustering along the Dead Sea fault.

Even if all the sections present a similar slip deficit, each segment underwent a different seismic history, which influences the likelihood of an earthquake to occur on each specific segment. Hence, in order to specify the areas where seismic hazard is higher, we computed a probability of earthquake occurrence on each segment following the probabilistic scheme proposed by Savage^52,53^ (complete description in the supplements). This model considers that the conditional probability of a future event can be estimated from the observed recurrence interval alone. However, these probabilities should be considered as indicative and only first-order calculations, as such model does not consider temporal clustering and all segments are considered independently for the computation.

Our model counts the number of inter-event periods that are shorter than the prediction window (time between the last event and the targeted date for probability calculation) and uses it to establish a probability of earthquake occurrence. The probabilistic model is Manichean, which introduces a bias for the interpretation of the probability; if the observed recurrence time is close but slightly longer than the prediction window, the probability will be rather low. Conversely, if the observed recurrence time is slightly shorter than the prediction window, then the probability will increase significantly. This is the case for example for the central Wadi Araba segment, where the probability of occurrence of an earthquake is substantially (30%) larger for the next 80 years than for the next 50 years (Fig. 3c). Hence, we consider that the entire confidence interval for the probability is more representative than the nominal probability to assess seismic hazard along the DSF. The probabilities of earthquakes along the southern DSF for the next 50 years and 80 years are relatively high everywhere, generally higher than 50%. This is in accord with the homogenously high-slip deficit presented previously and it supports the possibility to have clustering or single large ruptures on the DSF.

The new paleoseismological site of Taybeh enriches the data about the seismic history of the DSF. Moreover, we were able to estimate a slip deficit for the southern part of the fault, which has a critical implication for the assessment of seismic hazard. Indeed independently of any specific scenario considered, all the sections between Aqaba and South Lebanon present a similar slip deficit, which is quite large, at least 2 m, associated to a homogeneously high probability of earthquake occurrence. The irregular seismic activity presented by the earthquake catalogue and the fact that the fault presents a homogeneous slip deficit everywhere support the assumption of temporal clustering.

## Electronic supplementary material

Supplementary material
