# Supplementary material for: Slip deficit and temporal clustering along the Dead Sea fault from paleoseismological investigations
Source: Sci Rep. 2018 Mar 14;8:4511. doi: 10.1038/s41598-018-22627-9 (PMC5851983; doi:10.1038/s41598-018-22627-9)
Supplement: Supplementary file 1 — Supplementary material [file 41598_2018_22627_MOESM1_ESM.pdf]

Slip deficit and time clustering along the Dead Sea fault from paleoseismological investigations

Marthe Lefevre (1), Yann Klinger (1), Mahmoud Al-Qaryouti (2), Maryline Le Béon (3), Khaled Moumani (4)

(1) Institut de Physique du Globe de Paris, Paris, France,
(2) Jordan Seismological Observatory, Ministry of Energy and Mineral Resources, Amman, Jordan,
(3) Department of Earth Sciences - National Central University, Taiwan,
(4) Geological Mapping Division, Ministry of Energy and Mineral Resources, Amman, Jordan.

Supplementary materials

Table of content:

1. Units and fault zone descriptions 1
2. Description of earthquakes ruptures 4
3. Timing of events and Identification of historical earthquakes 6
4. Earthquake catalogue 14
5. Alternative scenarios 20
6. Probability computation 22
7. References 23
8. Table and figures 27

These supplementary materials consist only in technical descriptions and each part can be read independently of the others.

1. Units and fault zone descriptions

The trench is about 20 m long and 2.5 m deep. We can distinguish four main units, characteristic of different depositional environments (Fig. 1, Fig. S2). Each unit consists in a series of individual layers. The different strata consist in medium to coarse sand with few clasts and silts. The thickness of the layers varies from a couple of centimeters

to a few decimeters. The units tend to be more homogeneous and finer in the upper part, while sediments become coarser and more discontinuous in the lower part.
The central unit between marks 3 and 9 (M3-M9) is a reddish unstratified indurated sandy unit with gravels aligned vertically along fault zones (unit A, Fig. S3). This unit (A) has a structure similar to the one of the small push up located a hundred meters to the south of our trench and that brought reddish gravelly sand to the surface (Fig S1). In our trench, however, this structure has been partly eroded and does not reach the surface.
At the base of the trench there is a coarse unit (unit B), which contains two layers B1 and B2. The lowest layer (B1) consists of a conglomerate of matrix-supported pebbles, overlaid by a layer of indurated fine to medium yellow sand including some thin indurated clayey beds and gravels (B2). The conglomerate (B1) is not continuous laterally: it is visible only between marks M17 and M19, between M12 and M9 and at M2. Layer (B1) is thicker East of unit A, with a triangular shape thinning out eastward and unit A partly overthrusting on layer (B1). Although no obvious bedding could be seen in layer (B1), the compressional nature of unit A suggests that part of layer (B1) was dragged upward during successive activations of the push-up.
The unit Cu1 is characterized by channels, some of them flowing perpendicular to the trench, as shown by the lens shape of the gravel beds. The thickness of this unit varies across the trench exposure, to become thinner west of unit A. This variation may be due to the fact that water coming from the Jordan plateau is deflected by the local topographic barrier created by the unit A. The numerous channels in this unit are characteristic of an environment with significant precipitation and dynamic water flow, which could be associated to one of the major wet phases of the Holocene in ~ 5.6–3.5 cal kyr BP[1].
The upper 1.3 meters of the trench are formed by a series of sub-horizontal laminated sandy layers, beige to dark brown in color. Most of these units can be followed across the trench. These layers are labeled C to layer M (fig S2-S3).
Layers C to E are visible only on the eastern part of the trench. C is a thin layer of homogeneous brown sand, more humid than the surrounding units. Layer D is more heterogeneous, as it contains small lenses of gravels in the sand. Layer E is light beige and made of fine sand. Top of layers C-E is characterized by an erosional surface. Units Cu2 directly above this contact show local interfingering with coarser channel material in the middle of the trench (M9-13). This unit is made of finer material and becomes sub-horizontal in the eastern end of the trench. In some place we can distinguish three sub-layers in this unit (M16-17) but there are not continuous, and we consider them as one single unit. As for layers C to E, this unit is not visible in the western part of the trench. The layer F is a 10 cm-thick laminated beige layer. It is made of fine sand and few scattered gravels. The Layer G is a laminated coarse sandy layer. This layer is coarser on

the eastern part with gravels and pebbles and shows finer material in the central part (M9-16). On the western part of the trench this layer is thicker and it contains finer laminated sand. On top of G sits the layer H with an erosional contact between G and H. Layer H is brown in color, composed of sand with a large amount of coarse gravels. The layer I is a brown bedded sandy layer including laminas of clayish material white to dark brown in color. Layer J is a fine yellow sandy layer, which can be followed across the trench, although it gets thinner and discontinuous between M17 and M3. A thin discontinuous coarse layer characterized by small gravel lenses erodes into this layer. These lenses form the base of a thick beige laminated sandy layer, the layer K. This layer is well visible in the eastern part of the trench, but seems to be interrupted westward at M9. It is difficult to determine with certainty if it continues further west because of the lateral variation of facies. However, stratigraphic relations argue for a continuation of layer K between M1-M9. Indeed, the lower unit J is visible but discontinuous between M1 and M9, just below a thick unit coarser than the unit K on the eastern part. This coarser unit could be the continuation of layer K, acknowledging some lateral variation of facies that could be due in part to strike-slip motion along faults in a direction perpendicular to the trench. The lack of datable material in the eastern part of layer K prevented us from testing further lateral continuity of the units. At M9 units F to L are all affected by the fault zone, with the western side going down. A channel is nested at the toe of the scarp, which erodes into unit K between M9 and M3. This brown to dark brown channel unit (Cu3) shows large gravel lenses cross-bedded with sandy and more clayish beds. The layers L and M are the only traceable layers across the entire trench. The layer L is a laminated sandy layer containing few pebbles and gravel lenses. Its thickness decreases westward. This layer is erosive into layer K. The layer M is a fine to coarse sandy layer. It contains pebbles and small gravel lenses and clayey beds. In the western section few desiccation figures are visible.

The deformation is distributed over 15 m, with two main zones where it is larger. On the western end, between M3 and M9, faulting is mainly associated to the push-up structure formed by unit A. Cracks and fissures show opposite dip on each side of this structure. On the western side of this structure, faults are almost vertical with a slight dip eastward consistent with some minor thrust component of motion related to the push-up structure, in addition to dominant strike-slip faulting. On the eastern side of unit A, faults have a shallower westward dip. Although the stratigraphic position of unit A above unit B suggests that at some point these faults did accommodate some reverse motion in addition to strike-slip, according to obvious offset layers from layer C upward, these faults have mostly accommodated normal faulting during recent activation, in addition to the strike-slip. This motion is consistent with motion accommodated in the second main fault zone located between M16 and M18. In this fault zone, faults and

cracks dip westward with an average dip of 60°. In addition to strike-slip, these faults are responsible for down dropping the western part of the trench relative to the eastern part. The vertical motion mostly concentrates at M10 with at least about 30 cm of vertical displacement since 2000 years if we use unit F as a reference. This results in part in thickening of units westward.

As often, strike-slip motion is hardly recognizable in a fault-perpendicular trench as it results mainly in abrupt facies or thickness changes due to lateral motion. In our case, as our trench is located in quite an extensive bajada, these changes are even more difficult to see.

2. Description of earthquakes ruptures

The identification of earthquake ruptures is mostly based on consistent upward terminations of sets of cracks that vertically offset sedimentary units[2]. It follows the assumption that earthquakes ruptured the surface, thus we focus only on the large events. In the Wadi Araba the locking depth is estimated to be around 12 km[3,4]. Following the scaling laws of Wells & Coppersmith[5], event of magnitude 6.6 or above will break the entire brittle crust, which is comparable to the commonly used value of Mw=6.5[2]. Some smaller events could also be recorded in the trench, if they occurred close to the surface. For the coarser units, like conglomerates, it is particularly difficult to identify the exact location of some cracks termination. However, as coarser units are mostly located at the base of the trench, we consider that we recorded well the major events in the trench, at least for the most recent ones.

Eleven events have been unambiguously identified in our trench, and one additional event E5 is less constrained as it is associated to only 4 cracks (Fig S2). The older events, E9 to E11, are observed in the coarsest layers, which present erosive limits, thus deformation associated to these events in the trench is less pronounced than for more recent events. E11 is associated with cracks in the western part of the conglomerate (B1). As the contact with the layer deposited on top of it is erosive, we cannot see any vertical displacement, but cracks are well marked and underlined by rotated vertical pebbles. The presence of an erosive contact implies larger uncertainties on the age of this event, as the actual upper termination of cracks is unknown.

The event horizon associated to E10 is located on the top of layer B2. The evidence for this event is largely distributed and can be found both at the eastern and western ends of the trench. At M2 there is a small vertical displacement attributed to E10. The other cracks do not show visible displacements, which could be due to the erosive channelized units (Cu1) lying atop of layer B2.

The channels unit (Cu1) can be divided into two main layers separated by an erosional

contact, which follows the base of the channel lenses. This contact marks the event horizon for event E9.

The latter events broke and displaced the succession of sub-horizontal sandy layers. The associated deformation is more concentrated in two main zones, one between M18 and M16, the other at M9.

The event E8 is visible at M16, where the layers C and D are displaced downward (~3cm). Cracks associated to E8 are also observed within layer E, but the top of layer E is not deformed. Therefore, we consider that the event E8 occurred during the deposition of layer E.

The next event, E7, corresponds to a set of small cracks at M17. These cracks reach the top of the layer F, they are associated with a small vertical displacement. However, as the layer F is made of fine laminated sand the presence of cracks is unambiguous.

Based only on trench observation, one cannot exclude that the cracks associated to E8 and E7 correspond only to accommodation faulting produced by more recent earthquakes. Indeed some of them do not root deeply or seem to be shaped as a pull-apart structure, which weakens the evidence for independent events.

The event E6 is defined by a set of faults reaching the layer H, which is a thin, brown coarse layer. So it is difficult to see the end of the cracks. In addition, the base of this layer is irregular so we cannot identify any displacement. The cracks, however, are clearly visible in layers F and G, which are more laminated and made of finer materials.

Then event E5 is characterized by a limited number of cracks at M10-M9, they reach the top of layer I. In this brown layer there is a small intermediate darker bed, which shows a small vertical displacement.

The event E4 is not well marked in the trench. A series of cracks goes through the fine yellow sandy layer (layer J), but it is unclear if they stop at the top of layer J or if they also affect the layer above. Indeed, on the top of layer J sits a thin discontinuous layer of gravels where the deformation might be difficult to recognize.

The next event, E3, caused a major deformation in this area. There are series of cracks at M17 and at M9, which stop at the top of a layer K, a fine pinkish sandy layer, with almost no clasts. The deformation due to this event is clearly identified in the eastern part. At M9 the layers I, J, and K are clearly displaced downward (~25cm). The amount of displacement is actually larger than the displacement affecting layers directly above these units, attesting for a separate event. Its continuation in the western part of the trench is less clear because of the uncertainty on the identification of layer K at this place. But if we assume that layer K continues westward across the trench, although facies changes significantly, all cracks between M3-M7 reaching the base of the channelized unit (Cu3) would also match with the event horizon of E3.

The next event is associated to another series of cracks stopping at the base of layer L.

This series is located at M8 and M4 in an erosive channels unit deposited on top of the scarp of event E3, so the associated event, E3Supp postdates E3.

The event E2 is associated with a series of cracks located in the main zone of deformation (M18-M16). Its event horizon corresponds to the top of a sandy layer containing cobbles and some small gravel lenses (layer L). Between M17 and M16 this event produced an important vertical displacement (6-9cm), which is clearly visible in the layer I, K, and L.

The last event is marked by two sets of fractures located in the two mains zones of deformation (M18 and M9), these cracks reach the top of layer M, a light pink laminated sandy unit. Only minor deformation is associated to these cracks visible in the trench.

There is also a small group of cracks affecting only the unit A. As unit A eroded on the top, there is no offset associated to these cracks, but they are clearly identifiable through a succession of oriented gravels and pebbles.

3. Timing of events and Identification of historical earthquakes

Time constraints were derived from accelerator mass spectrometry (AMS) radiocarbon dating of detrital charcoals. The trench exposure presents a relatively rich and well-distributed amount of datable material. 32 charcoals coming from the two walls of the trench were dated (Tablesup 1, Fig. S4), we mainly used material from the southern wall, and few charcoals were collected on the other wall (charcoals 2, 9, 11, 17, 100, 119, 123) when there was no datable material on a layer in the studied wall. Only 4 samples (37,60, 71,79) had to be rejected, because their ages were significantly older than the other ages, relatively to their stratigraphic position. We interpreted them as likely reworked samples.

The time record is relatively complete and regular (Fig. S4) at least for the upper units, *i.e.* for the last 3ky. For the lower units in the trench, several erosive contacts have been identified. General continuity in our age distribution (Fig. S4), however, indicates that if we have hiatus in our trench, they have to represent only short periods of time, minimizing our chances to miss a major earthquake in our sedimentary record. The unit A is the oldest unit and dates back to 9 ky B.P., the layer at the base of the trench (B2) is dated at 8 ky BP. The chronological sequence is refined by combining a-priori information from stratigraphy relationship and ^{14}C ages in the Bayesian analysis approach of Oxcal[6]. According to the locations of the samples, phases have been defined to group samples that belong to the same unit and could not be clearly ordered based on stratigraphic position (Fig. S4).

The Taybeh trench exhibits evidence for at least 11 paleoearthquakes, and one more uncertain event. These earthquakes occurred during a period covering 8 ky (Fig. S5). We benefit from solid time constraints for the last 3 ky, which is also the period when historical and archaeological data are more numerous and accurate. However, no datable material could be found between the event horizons of E4 and E5, making the dating of this pair of events difficult because we cannot discriminate correctly these two events. The cracks affecting only the push-up structure are not dated, as the age of this unit is around 7000BC and this is the oldest unit of the trench. We only know that these cracks developed before the deposit of layers G and I, which are erosive on unit A.

In the following, each event in the Taybeh trench is identified by combining dating obtained using Oxcal and historical descriptions, which cover a wide region. All locations mentioned in this text are indicated on Figure S6. In the area, the historical reports are numerous and continuous in space and time. They give indications about the size of the earthquakes and thus contribute to constrain their lateral extent. For this area the seismites recorded in the Dead Sea are another source of information on earthquakes. Seismites are indicators of earthquakes occurrence in the area. They are intraclast breccia, which result from turbulences in the soft sediment[7] created by strong shaking. They record earthquakes from the whole Levantine area and not only earthquakes rupturing faults located in the proper Dead Sea basin. They are good markers to evaluate basin effects and recurrence patterns. We used seismites to support the existence of an earthquake and to constrain the epicentral distance of earthquakes from the Dead Sea[7].

The three older events are poorly constrained in age as datable material has been very limited. The conglomerates at the base of the trench includes only one sample (17) for which dating gives a wide age range. Likewise the events E10 and E9 are not well constrained as their event horizons are surrounded by thick layers containing only one or two samples, which give us only an approximation of the ages of the layers. Hence, the time intervals given by Oxcal are too wide to estimate properly an age. The age for E11, E10 and E9 are respectively between 6.1 ky BC and 5.4 ky BC, between 4.6 ky BC and 4 ky BC, and between 2.4 ky BC and 956 BC (Fig. S5). The poor dating associated with the lack of historical data for this time makes the matching of these events with specific historical earthquake impossible. We can only notice the vague mention of two events in 2100 BC and 1050/1070 BC in the area, more or less confirmed by archaeological remains[8,9] and the existence of seismites in the Dead Sea, which correlate with these two events[7,10].

The events E8, E7 and E6 are better constrained. Even if historical data about earthquakes during the corresponding periods are scarce, we can still propose a specific matching historical earthquake for each of these events:

The event E8 is dated between 885 BC and 514 BC (Fig. S5). This time interval is wide but covers only few documented events, although only one is described in details. In 759 BC an earthquake produced great destructions and many casualties in Judea, Samaria and Galilee[11]. Zachariah gave around 520 BC (i.e., ~240 years later) a detailed description of the event, where he described a large landslide developing on the Mount of Olives, southeast of Jerusalem, without a clear origin. He suggests that it could be due to an earthquake[9]. All the descriptions of this event concern the north of the Dead Sea. Ferry et al.[12] also reported an event in a trench in the Jordan Valley that they associate to the 759 BC event. Finally, in their study of cores from the Dead Sea Kagan et al.[7] identified two events on the mid 8th century BC. Historical information about an event in this period refers mainly to the Jordan valley event, but the event E8 could be the second event observed by Kagan et al.[7], even if it remains uncertain in view of the scarce data.

The event E7 is modeled between 520 BC and 280 BC (Fig S5), as for E8 the time interval is wide and very little information about events in this period is available. We could correlate this event with a down-faulting event along the northwestern tip of the Gulf of Aqaba, which is recorded by a buried coral-reef platform[13,14]. This event has been dated using 14C and U-series on coral and yields ages of ~2.4 ka BP, which is in agreement with the seismic period of E7. Moreover in the Qatar trench[15] an earthquake is identified for a period partly similar between 313 BC and 213 BC. It seems that the event E7 ruptured the central and southern part of the Wadi Araba. The northern limit remains uncertain, but as there is no indication of an event in the 4th century BC in the cores of the Dead Sea basin[7,10] it seems that this event stopped before the Dead Sea basin, probably in the area of Taybeh.

The time interval associated to the event E6 is narrow, between 160 BC and 117 BC (Fig. S5). This period is compatible with a seismite layer dated about mid-2nd century BC that was consistently documented all around the Dead Sea basin, and was associated to a large magnitude event, which would have affected the northern part of the Dead Sea fault[7,10]. We cannot really conclude about this event, as there is absolutely no mention of an earthquake in the southern part of the area. This event might actually be a small event triggered by a larger one, which occurred north of the Dead Sea, or could have ruptured the northern part of the Wadi Araba and be amalgamated with another event. In this cases the fact that the Wadi Araba is relatively sparsely populated contrary to the Jordan Valley would explain why there is no historical report about this small event in this region.

Events E5 and E4 are recorded in two successive layers in the trench, which are not

dated, thus they are associated to the same period. E4 and E5 are modeled respectively between 80 BC and 141 AD and between 14 BC and 205 AD (Fig. S5). Around the beginning of this era two events are described in the region of the Dead Sea, Judea and Wadi Araba, the 31 BC event and the 114 AD event. The 31 BC event is only described in Judea, during the Jewish War by the historian Flavius Josephus[11]. However, the damage seems greatly exaggerated, with a number of casualties larger than the actual population of the region[9]. It has been proposed that the 31 BC event caused the abandonment of Qunram, but without substantial proof[11]. Ruptures associated to the 31 BC event were not observed in the Jordan valley[12], and there is no archaeological evidence of this earthquake in Judea. Apart from Josephus there is no descriptions of any damage in the area, so this event may have occurred in the Wadi Araba, which would explain the weak effect north to the Dead Sea. There is no event recorded for this period at Qatar[15], which suggests that it ruptured the northern part of the Wadi Araba and potentially the Lisan peninsula segment too. This is consistent with the observations of Kagan et al.[7], who described an intrabasin seismite for this event. They locate the 31 BC event in a radius of 40 km around the Dead Sea.

The 110-114 AD event is attested only by archaeological data and is quite disputed. Indeed damage is described in Petra, Masada, Avdat and several other sites along the Petra-Gaza road[9,16]. Nevertheless there is generally no direct evidence to suggest that the damage is due to an earthquake and it has been proposed that they might result from wars. There is one excavation site in Masada where the rooms seem 'filled with the result of an earthquake[9,17]. The presence of a series of crack dated between 14 BC and 205 AD in the trench at Taybeh, i.e. at the same latitude as Petra but in the valley, could argue in favor of an earthquake. The fact that there was only moderate damage could indicate that this event was of limited magnitude. This event occurred around Taybeh, potentially southward as there is no indication of an event at this time in the Dead Sea cores. As this event is not observed in the Qatar trench[15] it had to stop on the north of the extensional jog of Yotvata. In the light of the archaeological data, we consider that E5 could correspond to the 31 BC event and onccured on the section between the Lisan peninsula and Taybeh and E4 might be the 110-114 AD event and ruptured the central Araba section. Nevertheless as there is little evidence for E5 in the trench, this event remains uncertain and these cracks could also be associated to E4. Therefore, if one considers that only E4 exists, archeological evidence suggests that E4 would be the 31 BC earthquake.

The event E3 is dated between 287 AD and 815 AD (Fig. S5). This time window is wide, as the sandy layers I, J, K and L lacked datable organic matter. During this period two events are described in the area south of the Dead Sea: the 363 AD event and the mid 8th century AD crisis. We can also notice the occurrence of several earthquakes during the

7th century, but they are mostly described further north.

During the night of 363 May 18th two earthquakes occurred in the Levantine area. As they are coeval it is difficult to separate the effects of the two shocks. Nevertheless it seems that the second shock affected more the southern part of the region. A seiche was reported on the southeastern coast of the Dead Sea, as well as much destruction in southern Palestine localities and in the city of Petra[9]. Moreover, destructions of Roman buildings are documented in Aila (Aqaba), which are well time-constrained through dates of more than 100 coins found under collapsed walls, to be post 360 AD[18]. A seismite of the Dead Sea could be unambiguously associated to this event[7]. As the 363 AD earthquake is visible in the Qatar trench, Klinger et al.[15] proposed that this event ruptured at least the southern section of the Wadi Araba, between Qatar and the Gulf of Aqaba, the northern limit of this rupture remaining unknown.

During the 7th century AD three earthquakes were reported. One event occurred in 634 AD, which is described in Palestine, mainly in Jerusalem[9], arguing for a location close to the Dead Sea. The other two events occurred in 659/660: the first event occurred in June and it affected the Palestine and many places in the Jordan Valley. The second, in September, destroyed a great part of Jericho and several monasteries in the hills, East of Jerusalem[11,19]. Although these events are mostly described based on reports from locations further north and around the Jordan Valley, the September 659 AD earthquake was felt in a narrow area surrounding Jericho. Thus we proposed that this event occurred along the Jericho segment. In fact, the June 659 AD event was located by Langut et al.[20] in the northern Jordan valley, in the vicinity of Beit-shean, which would be consistent with the 7th century event observed in the 3D trench by Wechsler et al.[21]. Eventually, Haynes et al.[22] have reported evidence of damaged water cistern in Tilah, which are dated to the the 7th century, and might correspond the 634 AD event.

The mid 8th century AD event is confusedly described and it has been shown recently that descriptions of different earthquakes were often aggregated into a single giant earthquake; Ambraseys[9] demonstrated that at least three large earthquakes struck the region south of the Lebanese range between 746 AD and 757 AD. And although damage is reported for many of the main cities of the region, it remains difficult to ascertain epicentral locations for each of them based only on macroseismic data. One large event in 749 AD took place in the Jordan valley and reached the north of the Sea of Galilee, as shown by paleoseismological and archaeological studies[12,23-25]. At Qatar Klinger et al.[15] identified another event around the mid 8th century AD in the Wadi Araba. Moreover there is also evidence for damage in the ancient cities of Aila[18] and Petra[26]. In the trench at Tilah there is an event dated between the 7th and the 11th century AD[22]. The authors propose that this event correspond to the 873 AD earthquake. Nevertheless this earthquake is described only in Medina, which is more than 1000 km away. If the event

observed in Tilah is the 873 AD event, it has to be a very large event to be felt in Medina and in this case it would have to be felt also in closer cities. In view of the absence of historical reports about a 873 earthquake in Wadi Araba or Jordan Valley areas, we assume that this event corresponds to the 8th century AD event, which is mentioned in many places in the area, rather than the 873 AD event. Thus the mid 8th century AD event might be recorded in Tilah, in this case there would be evidence of a 8th century AD events all along the Wadi Araba, which argues for a correlation between E3 and one earthquake occurring during this seismic crisis.

If we consider that E3 correspond to a 7th century AD, like the one observed in Tilah[22], it implies that E3 occurred on the section between Taybeh and Tilah in the 7th century and two earthquakes occurred in the Wadi Araba during the 8th century AD, one between the Gulf of Aqaba and Qatar[15] and the other north of Tilah. While if we consider that E3 correspond to a 8th century AD event, it implies that the 7th century AD event observed at Tilah correspond to an earthquake having ruptured the section from Tilah to the Lisan Peninsula, and the 8th century AD crisis affected all the Wadi Araba.

We favor the second scenario and consider that the event E3 corresponds to a 8th century AD event, as it implies a smaller number of events. Moreover it is more consistent with the historical data, which point to a location further north for the 7th century events[9,11]. It signifies that the 363 AD event did not reach the site of Taybeh.

The event E3Supp is modeled between 819 AD and 1395 AD, a period during which three earthquakes are described in the area: the 1068 AD event, the 1212 AD event and the 1293 AD event.

In 1068 AD there were two events[9,27], the 1068 March 18 event, most probably located south of the Dead Sea, and the 1068 May 29 event, located closer to northern Palestine, which have been aggregated into a single giant earthquake in most of the early historical accounts[12]. During the March 1068 event almost the entire population of Aqaba was killed and damage to mosques was reported as far as Medina and Cairo[9]. Furthermore modifications of water springs are reported close to the city of Tabuk[9]. There is evidence of surface ruptures associated to the 1068 event in the Avrona playa, close to Eilat/Aqaba[28,29]. This event is located by Klinger et al.[15] between the site of Qatar, where there are observations of the event, and the Gulf of Aqaba. A magnitude M_W 6.5 or higher has been proposed using the length of the rupture[5], which is in good agreement with values proposed earlier, based on macroseismic reports and geomorphological observations[28-30]. If we consider that the event E3Supp is the 1068 AD event, it means that the 1068 AD event ruptured at least the section between the Gulf of Aqaba and Taybeh, *i.e.* a section 110km long. This rupture length corresponds[5] to a magnitude of 7.4 or higher, which is too large compared to the macroseismic reports. So we assume that the event E3Supp visible at Taybeh is not the 1068 AD earthquake.

The 1212 AD event is manly described in the south Levantine area. This earthquake produced extensive damage in the city of Aila (Aqaba). It is also reported in Egypt, the Monastery of St Catherine in Sinai was partly destroyed and the shock was strongly felt in Cairo where several houses collapsed. Destructions were also reported in north of the Wadi Araba, in Al-Karak and Al-Shobak on the Jordanian plateau[9]. Based on the damage reports and geomorphological observations this event is considered as an important event of about M_w 7.5[7,10]. This event is clearly identified in the Dead Sea cores[7] as well as in the Qatar trench[15]. It has been proposed that this event occurred along the segment between Qatar and the Gulf of Aqaba and potentially in the Gulf as well[9,15].

The 1293 AD event is mainly described in Karak, where it produced great damage in the citadel. This event was also felt in Ramla and on the Palestinian coast from Gaza to Qaqun[27,31]. There are seismites in different places in the Dead Sea where this event is clearly identified[7,10]. This event is usually located on the eastern flank of the Dead Sea, its southern limit is not defined accurately.

Regarding the ages the event E3Supp may correspond to the three earthquakes, nevertheless the 1068 AD event is felt only on the southern part of the Wadi Araba and if we consider a rupture from Taybeh to the Gulf of Aqaba it implies a larger earthquake than it seems to be as regards to the historical descriptions. The 1212 AD and 1293 AD events were both felt close to Taybeh, but following the damage reports the 1212 AD event was bigger further south. Indeed most of the damage is recorded around the Gulf of Aqaba, as St Catherine, which is much further south in the Sinai Mountains, and present an important degree of destruction. This is more in agreement with a rupture in the Gulf of Aqaba than in the Wadi Araba. Kagan et al.[7] based on the seismites analysis propose a distance of the epicentre from the Dead Sea for several earthquakes. In the case of the 1212 AD event they propose a relatively large distance of about 250-300km, which support the location of this event in the Gulf of Aqaba. That is why we propose that the event E3Supp is the 1293 AD earthquake, which implies that this event ruptured the section extending north of Taybeh, and 1212 AD ruptured from the Gulf of Aqaba until Qatar.

The penultimate event, E2, is modeled between 1448 AD and 1714 AD (Fig S5). During that interval, the only significant event reported in the area is a magnitude M6-7 event in November 1458[9]. The main destructions related to this earthquake are in Jerusalem and Al-Karak. There is no mention of this event neither in the Wadi Araba nor in Aqaba. Nevertheless this event is recognized in most of the seismite sequences cored in the Dead Sea[7,10], based on the seismite they proposed that the 1458 AD event occurred relatively close to the Dead See, around 50 km away. It is observed in the trench at Qatar[15], it may also be one of the events recognized in the cores at Aqaba[32]. The rupture did not reach Aqaba, otherwise the city would have been more affected. The northern

limit of this rupture remains uncertain as there is not any clear evidence of this event at Tilah. Indeed in Tilah the most recent event is dated between 1155 AD and 1918 AD[22], it could correspond to the 1212 AD, 1293 AD, 1458 AD, 1546 AD and 1834 AD events. The 1212 AD event seems to have occurred further south than the other regarding the damage reports. The 1546 AD event has been firstly described as a major earthquake in many places in the north Dead Sea (Safed, Tiberias, Ramla, Jerusalem, Hebron, Karak, and Nablus)[8]. These descriptions seem highly exaggerated it would rather be medium-size event affecting mainly Jerusalem[9]. This is in agreement with a rupture located in the area close to Jerusalem, probably along the northernmost west Dead Sea shore. The 1834 AD event caused damage in Jerusalem, Bethlehem, Madaba, Jaffa and Caesarea, the associated area is large and make difficult to locate its epicentral region[9], nevertheless most of the sources are located North of the Dead Sea, so we consider that this event occurred in the Jordan Valley. The descriptions of the 1293 AD and 1458 AD event are located in the same area, so these descriptions are not discriminant to determine which earthquake correspond to the last event visible at Tilah. Nevertheless we identified the 1293 AD event in the Taybeh trench, as it is not visible in Qatar that suggests that its rupture extends north of Taybeh. Moreover it as been proposed that this event was located close to Karak, the most affected city, in the northern Araba[9,26]. So the last event visible at Tilah might be the 1293 AD earthquake, it implies that the 1293 AD event ruptured the section from Taybeh until the Lisan Peninsula, while the 1458 AD earthquake stopped further south, probably at Taybeh. Based on the descriptions of the destructions and the paleoseismological observations all-together we proposed that the event E2 is the 1458 AD earthquake, which ruptured a section of the Wadi Araba fault located between the site of Qatar and our site.

The last event is modeled between 1688 AD and 1799 AD (Fig. S5). Agnon[26] proposed that the 1834 AD event occurred in the Wadi Araba and would correspond to the last event observed at Tilah. Hence, regarding the dating the event E1 might be the 1834 AD event. Nevertheless the descriptions of the 1834 AD event consistently locate this event north of the Dead Sea and based on these reports we would favor a location in the Jordan Valley area for this event.

For the 18th century there is no other major earthquake described in the south of the Levant area. If there had been a large event during this period in the Wadi Araba, as it is quite recent it should have been recorded in historical reports. Thus we consider that the event E1 is a smaller event, likely a Mw~5 event, and since there is no large city in this region, this event did not produce significant damage and was not described. Small events can locally be recorded in trenches if the trench is close to the epicentre and if they produced a surface rupture[33]. In the catalogue we do not take this event into

account, as it is small and cannot be considered for the estimates of earthquake recurrence.

4. Earthquake catalogue

In order to investigate the fault behavior and the regional seismic hazard along the southern Dead Sea fault, we established an earthquake catalogue for the part of the Dead Sea fault between the Gulf of Aqaba and the southern limit of Mount Lebanon. The catalogue is built by combining data from the literature (historical data[11,26,27,31,34-37] and paleoseismological data[12,15,22,25,29]) and paleoseismological results presented in this work.

To build the catalogue we firstly determine a simplified geometry of the fault, which describes the sections rupturing during an earthquake. The geometry we choose contains 9 segments that are described hereafter: The Wadi Araba section is split in three segments separated respectively by an extensional jog, the Yotvata playa, and a compressional jog at Jabal al-Risha. Similarly, the Jordan Valley section is also split in three segment: One segment parallels the western bank of the Dead Sea and ends just north of Jericho. The central segment stretches between Jericho and the Beit-She'an Valley, which is formed by the intersection of the Jordan valley fault (JVF) and the Carmel-Gilboa Fault System (CGFS), which branches westward from the JVF. The exact location where to end this segment in the Beit-She'an Valley is debatable, as the JVF and CGFS interact over a several kilometers long zone. In any case this zone is limited to the north by the northern margin of Beit-She'an Valley. The last segment extends from the Beit-She'an Valley to the northeastern shore of the Sea of Galilee, where the Jordan fault interacts with the Jordan Gorge section[38]. North of the Sea of Galilee we defined two segments, the Jordan Gorge segment and the Hula basin segment.

In a second step we matched past earthquakes with fault segments:

In the Wadi Araba for the southernmost segment, between Aqaba and Yotvata, we used the catalogue proposed by Klinger et al.[15]. For the central and northern segments we used mainly the data of the Taybeh trench, the detailed description of the location of paleo-earthquakes on these segments is described above in this study.

Along the Jericho segment, we were able to relocate several events until the fifth century: The most recent one is dated from July 1927. It was the first significant event (Mw=6.3) in the region to be recorded worldwide by seismometers. These records

allowed assessing the epicentral location[39,40], which is on the western side of the Dead Sea, close to Mitzpe Shalem[39]. Another recent earthquake might have occurred on this segment in 1834 AD. The 1834 AD event is mainly described north of the Dead Sea especially in Jerusalem where several minarets and churches collapsed. Damage associated to the 1834 AD earthquake was also reported in the cities of Bethlehem, Madaba, Nablus, Caesarea and in the Dead Sea basin where asphalt floated[9]. Distribution of seismite in the Dead Sea basin argues for an epicenter rather close to the basin[7]. Hence, this event occurred in the Jordan Valley, very likely along the Jericho segment. The historical descriptions of the 1546 AD event seem to indicate a similar intensity map to the 1927 event, hence we locate the 1546 AD event on the same segment. The May 29th 1068 AD earthquake is known mostly to have destroyed al-Ramla. Only slight damage is reported in the other places in the region. This event is mentioned briefly in Jerusalem and Banias. Hence, the scarcity of historical descriptions and their heterogeneities suggest a possible exaggeration of the damage. The area of distribution of the damage linked to this event points to a location in the Jordan valley. Ferry et al.[12] see only one event at this time in their trench, which is located in the central part of the Jordan valley, and they suggest that it corresponds the 1033 AD event. Thus we assume that the 1068 AD event occurred on the Jericho segment. However, owing to the lack of historical data for this earthquake, its authenticity remains arguable[26,31] and if it existed magnitude might have been smaller than previously proposed. Thus for the scenario considering the smallest extent for each earthquake (in light blue on fig 3, S7-S12) we attributed a magnitude of 6.5 to this event. All the reported damage caused by the September 660 AD event were located on the western flank of the Dead Sea, Jericho was partly destroyed as well as several monasteries in this region, including St. John the Baptist monastery. Thus we assume that the September 660 AD earthquake occurred along the Jericho segment. This is also supported by the presence of seismites in at least two cores drilled in the Dead Sea[7,10]. Another event dating back to 419 AD has been described in this area. However the available testimonies are less specific, several Palestinian villages have been allegedly destroyed, indicating that this event occurred along the Jordan valley. We favored the Jericho segment since Kagan et al.[7] described it as an intra basin seismite, implying that it took place close to the Dead Sea basin.

Along the central Jordan Valley segment, we used mainly the results of Ferry et al.[12] to determine the chronology of earthquakes, except for the most recent events since the upper part of their trenches are plowed soils. The 1033 AD event appears on all four trenches both north and south of the segment[12]. It was felt in Jerusalem – where according to Ibn Al-Jawzi (A.D. 1113-1200) the walls were damaged while still in construction - Ramallah, Jericho, Nablus and Tiberias and throughout Judea and Syria[41].

Hence, we assigned this earthquake to the central part of the Jordan Valley fault, although we could not rule out that it could also have ruptured the northern part of the Jordan valley, in view of the macroseismic data. The second event recorded in Ferry's trenches dates from 749 AD. Great destructions and many casualties have been reported in Judea, Samaria and Galilee at this time[11]. The mid 8th century is known in the Dead Sea area as a period of intense seismic activity and historical descriptions tend to merge multiple events together. Since this earthquake is also recognized in a trench north of the Sea of Galilee[25], we consider that it ruptured all three segments (the central and north Jordan valley segments and the Jordan Gorge segment).

Along the northern Jordan valley segment, the most recent event we identified is the 854 AD event. This earthquake caused several landslides around Tiberias, and many casualties in the city due to collapses[9,11]. Before this event, the 749 AD event ruptured all the Jordan valley section. Another event described in this region is the 659/660 AD earthquake, which was followed by a large series of aftershocks. This event was reported in several places across Palestine[11,20,31]. There is also some debatable evidence of an earthquake proposed at Gerasa and Pella[16]. This report indicates a location of the earthquake in the Jordan valley. We assume that it occurred on the northern segment because there is no evidence of an event in the trenches on the central segment[12]. Furthermore Wechsler et al.[21] describe an event around that time at a site on the southern part of the Jordan Gorge in Beteiha. They propose that this event corresponds to the 660 AD event, which corroborates a rupture in the northern area of the Jordan valley. This earthquake would have ruptured the northern Jordan valley segment and the Jordan Gorge segment.

The Jordan Valley section of the DST is interpreted to have ruptured during the March-363 AD earthquake based on historical records[9]. The historical descriptions cover a wide area, which extends from Banias in the north to Areopolis in the south. The southern limit of the area where this earthquake was felt is not well constrained because of the occurrence of another earthquake in the Wadi Araba the same year that lead to some confusion in descriptions. The 363 AD event is not clearly identified in the trenches on the central Jordan valley segment[12], nevertheless we can notice that there is one event in these trenches dated before the 6th century AD, but the time constraint is weak and it is not possible to conclude about the age of this event. As most of the site that where damage during the 363 AD event are located west of the Jordan valley, it has been proposed that this earthquake occurred on the Carmel fault[26]. However such asymmetry, which is observed for several events in the area, might also in part be attributed to the difference of density of population between the Eastern and the Western Levant. Moreover Wechsler et al.[21] identified in the Beteiha trench displacements associated to three events occurring on the 4th century AD. The authors

assume that one of these events could be the 363 AD event, so the 363 AD-earthquake rupture reached as far north as the Beteiha valley. Based on this observation we proposed that the 363 AD event ruptured the Jordan Valley fault. The southern limit remains unclear, but in view of the historical report distribution this earthquake was quite important and ruptured at least the northern segment of the Jordan Valley and most probably the central segment too.
In addition to these events that are well described, there is also a series of events loosely described during the 12 century, which are associated with the Jordan valley area. In 1113 AD the Christians in Jerusalem report two events occurring in the Kingdom of Jerusalem (which lies from south to north between the northern Dead Sea basin to the Sea of Galilee), likewise in 1114 AD an earthquake is described by authors living in Jerusalem but as there is no indication of damage in Jerusalem, Ambraseys[9] proposed that it occurred around the Sea of Galilee. Finally, an event is described in Jerusalem in 1117 AD. All the reports of these events are vague and do not indicate significant damage, so we assume that between 1113 AD and 1117 AD a series of smaller earthquakes (Mw ~6) occurred all along the Jordan Valley.

The last main section, north to the Sea of Galilee is divided in two segments. The Jordan Gorge Segment, which lies between the Sea of Galilee and the Hula basin. The other is the Hula basin segment, which constitutes the western flank of this basin and stops south of Mount Lebanon where the fault divides into two faults, the Roum fault to the west and the Yamouneh fault to the east. These two segments are short ~20km compared to the others.
Along the Jordan Gorge segment, two 3D paleoseismological studies[23,25] and one archaeological sites[42,43] provide indications about earthquake history. The 3D trenches provide us with lateral displacement measurements across the fault. These displacements correspond to co-seismic displacements associated with well-known historical events, but a part of the displacement could also be due to aftershocks or local events that were triggered by larger regional events. The 3D trenches show a dense chronology of earthquakes[25]. The most recent earthquake is the 1759 AD event, which severely affected Lebanon. Two earthquakes actually struck the area in 1759 AD. The larger shock on November 25th destroyed all the villages in the Beqaa valley. Baalbek was ruined. Safed, Ras Baalbek, and Damascus were damaged, and the earthquake was felt as far as Egypt and Anatolia, 1100 km away[44]. A smaller earthquake occurred about one month earlier, on October 30th 1759. This shock ruined Safed, Qunaitra, and many villages nearby, and triggered a seismic seiche in the Sea of Galilee[8]. This later event is also described at the Crusader castle of Vadum Jacob[42,43] where it is associated to 0.5 m of displacement, which is confirmed by the measurement in 3D trenches by Wechsler et

al.[21]. Studies on the Rachaya fault[45] and on the Serghaya fault[46,47] showed that the two 1759 AD earthquakes occurred along these faults. So, the 1759 October earthquake ruptured the Jordan Gorge segment and the Rachaya fault, while the November event ruptured the Serghaya fault. The previous event recorded is the 1202 AD event, which shook mainly Lebanon and western Syria. The cities of Baalbek, Nablus, Acre, Safed, Tyre, Tripoli, among others, where severely damaged. Shaking was felt throughout the Middle East[44]. This event is also observed in the paleoseismological trench of Yamouneh[48], and at the Crusader castle of Vadum Jacob[42,43]. This earthquake occurred on the Yamouneh fault, the Hula basin segment, and the Jordan Gorge segment. In the Beteiha site, Wechsler et al.[21] observe 2.1 m of displacement associated to this event, such as Ellenblum et al.[43] in Vadum Jacob. The 3D trench also exposes evidence for a 1.2 m displacement linked to the 749 AD earthquake[21]. This event is known to have ruptured the Jordan valley section[12], and the presence of surface rupture associated to this earthquake north of the Sea of Galilee implies that this event extended further north than expected and the Jordan Gorge segment correspond to the northern limit of the rupture. We consider here that the 749 ad earthquake reached the Beteiha valley but do not ruptured all the Jordan Gorge segment as there is less mention of this event in Lebanon. There is possibly an event during the 7th century AD that is recorded in the trench at the Beteiha site in the Jordan Gorge. The occurrence of this event remains unclear, as the associated displacement is small (almost non-existing). We assume that this 7th century event corresponds to the June 659 AD earthquake, which occurred on the Jordan valley section and extended a little north. Another event with a displacement about 1.2 m is recorded in the 3D trench, the displaced deposits give an age for the seismic event between 505 and 593 AD. During that period several earthquakes caused damage in the region, but mostly on the coastal area. It is the case for the 502 AD and 551 AD earthquakes. Paleoseismic studies point to the offshore Lebanese thrust system as the most likely source of the 551 AD earthquake[49]. So we could tentatively associate the displacement in the trench with the 502 AD event, even if an offshore source appears more consistent, as this event mainly destroyed Acre, Sidon, Tyr, and to a lesser degree Beirut. In that case, the 502 AD earthquake would have ruptured the Jordan Gorge segment, the Hula basin segment, and the Roum fault. Moreover, as the 4th-8th century AD period is well documented historically, chances that an additional historical event, with more than 1 m of slip, went unnoticed is unlikely[35,37]. In the 3D trench at the Beteiha valley the oldest displaced channel records evidence for six additional earthquakes. Two of them affected the upper units with additional cumulative slip of 1.8 to 3.6 m. Both are likely moderate in size, each with 0.9-1.8 m of horizontal slip. Three of the six earthquakes were dated in the 4th century AD, including the two events that affected the upper units. The authors proposed that it is the 303 AD, 348 AD and 363 AD

events. The 303 AD event caused damage mostly on the southern Lebanese coast (Sidon and Tyr). The 343 AD event is described in Beirut, where a large part of the city was ruined. The 343 AD event could also be associated to the penultimate event visible in the Yamouneh trench, which is dated between 30 BC and 469 AD[48], which could explain why most of the reports are further north. However, in this case the rupture length of this event is quite important as it extends from the Jordan Gorge to the Yamouneh fault, just as the 1202 AD earthquake, which is in disagreement with the limited available historical reports. The 363 AD event is known to have ruptured the Jordan Valley section, as most of the historical reports for this event are in Palestine and around the Jordan Valley. The presence of a rupture in the Beteiha trench implies that the rupture extended also a little north of the Sea of Galilee. The ages of the three other events are not well constrained, two of them are dated from the 2nd century. Associated displacements are not clear and in any case quite small. One of them could be associated with the 130 AD earthquake, which caused damage at Caesarea, but the reports are very unclear even about the location of the city. Finally, the oldest event visible in the trench is associated to a large displacement and dated between 392BC and 91 AD. Ellenblum et al. 2015[50] find evidence of a coseismic offset under the castle of Vadum Jacob, reaching ~2.5m dated to circa 142 BC. So we propose that the oldest event at Beteiha could be the 140 BC event, which was described along the Lebanon coast[8,9]. This event was associated to a large magnitude[8], which is in accord with the measured displacement. Ellenblum et al. 2015[50] also point to a post Hellenistic event, with an abandonment of the site around the mid-first century BC, for which we do not propose a specific earthquake correlation.

The Hula basin segment is small and located between the intersection of the Roum and Yamouneh faults in the north and the intersection of the Rachaya and Jordan Gorge faults in the south. This segment behaves like an inter-segment due to its particular location, *i.e.* this segment breaks only with others surrounding segments of the fault. As a result, in order to identify the events that occurred on the Hula basin segment we firstly investigated earthquakes occurring on the Roum and Yamouneh faults. On the Roum fault only one event is well identified during the historical times. Nemer et al.[51] dug a trench on the Roum fault and identified an event that occurred after the 3rd century AD, based on historical reports it could be the 551 AD or 1837 AD event. As the 551 AD earthquake is well located offshore of Lebanon[49], they assume that it is the 1837 AD earthquake. Destructions or heavy damage caused by this earthquake extended along a relatively narrow zone from the coastal area of Sidon to the Sea of Galilee. Sidon was the most damaged location. Damage was also reported in Beirut, along the Bekaa valley, at Banias, the area of the Sea of Galilee is also affected. South to the Sea of Galilee the damage is weaker. We assume that the 1837 AD earthquake ruptured the Roum fault

and the Hula basin segment. The Hula basin segment also ruptured during the 1202 AD earthquake, for which there are surface ruptures attested on the Yamouneh fault and on the Jordan Gorge fault. The 348 AD event, which is described in the Beteiha trench, seems to have ruptured the section coming from the Yamouneh fault, so it must have ruptured also the Hula basin segment. The last event we located along this segment is more uncertain, namely the 198 BC event. At this time there was a series of shocks felt in the region of Sidon, and almost two thirds of the city collapsed. It could correspond to the second event described in the trench on the Roum fault[51], although as the time interval associated to this event is wide, reaching conclusion remains hazardous. In this case the 198 BC might have ruptured the Roum fault and the Hula basin segment.

5. Alternative rupture scenarios

For several events described previously we have seen that uncertainties remain about their extensions and limits, or even their locations. In the precedent sections we have presented the historical and paleoseismological data about past earthquakes available in the area, and then our favorite scenario. Here we will focus on possible alternatives for this scenario and evaluate the impact on our output model.
Most alternatives deal with the Wadi Araba, and the identification of historical events at Taybeh and Tilah. We present only one change concerning solely the area north of the Dead Sea.

Alternative 1: the northern limit of the 363 AD and 749 AD ruptures along the Jordan Gorge segment is located further north
Our favorite scenario considers that the 363 AD and 749 AD earthquakes, observed in the Jordan Valley trenches and widely reported in this area, ruptured the north Jordan Valley section up to Beteiha, but not the entire Jordan Gorge segment, as they are described in the 3D trench by Wechsler et al.[21] but not in the Vadum Jacob site[50]. In figure S7 the slip deficit is calculated considering that these events ruptured also the Jordan Gorge section. This change implies a slightly smaller calculated slip deficit in the Jordan Valley although it does not impact our conclusion. Indeed, for the 749 AD earthquake we always used the co-seismic displacement measured in trench[21] to assess the lower slip-deficit value.

Alternative 2: effect of the location of the northern limit of the 363 AD rupture

At Taybeh we propose that the event E3 correspond to a 8th century event, which implies that the 363 AD event does not reach Taybeh. We previously limited the rupture of the 363 AD event to the southern Wadi Araba segment. Nevertheless as strike-slip ruptures do not always stop at fault discontinuities[52], we can wonder if this earthquake extends further north. In figure S7 we consider that the 363 AD event ruptured the southern and central segments of the Wadi Araba, and stopped at the southern limit of the Risha compressional jog. This modification reduces the slip deficit the central Araba segment to 0.7 m and leads to relatively small values of slip deficit in the Wadi Araba.

Alternative 3: the event E3 is associated to a 7th century AD earthquake

Another debatable point is the identification of E3 as a 8th century AD event rather than a 7th century AD event as in Tilah. Indeed in Tilah Haynes et al.[22] described two events during this period, one in the 7th century AD and another between the 7th and the 11th century AD, whereas there is only one event recorded at Taybeh for this period. We firstly favor a scenario with a large 8th century AD event in the whole Wadi Araba and a small 7th century AD event in the north. In figure S8 we present a scenario with a 7th century event between Taybeh and Tilah and two 8th century AD events. This scenario leads to very large slip deficits in the Wadi Araba, up to 7.2 m. Accommodating such large slip deficit in one earthquake implies very large magnitude (M>>7.5) earthquake, which have not been documented along the DSF so far.

Alternative 4: combination of alternatives 2 and 3

The previous two alternative scenarios have opposite effects. Thus we tested both changes together (figure S9). We observe that the lengthening of the 363 AD event is not enough to compensate the shortening of the 8th century AD event. That support again the fact that very large earthquakes occur occasionally.

Alternative 5: effect of the location of the northern limit of the 1293 AD earthquake

Another debatable point is the limit of the 1293 AD event. We previously considered that the 1293 AD event was visible at Taybeh and Tilah, thus it would have ruptured the section from Taybeh to the Lisan Peninsula. Nevertheless, the dating of the events at Tilah allows for another interpretation, with a 9th century AD event. We present in figure S10 an alternative of our scenario with a 9th century AD event located on the Lisan Peninsula segment and the 1293 AD event, which is recorded at Taybeh, to rupture the section between Taybeh and Tilah. This scenario increases significantly the slip deficit on the concerned segments. This supports the fact that the 1293 AD earthquake ruptures both sections.

It is note worthy that if we consider that the 1293 AD earthquake ruptured the area from Taybeh to the Lisan Peninsula and that there is an additional event on the Lisan Peninsula during the 9th century AD, the slip deficit would decrease slightly. For the Lisan Peninsula section the slip deficit is then of 0.9 m (this scenario is not represented).

Alternative 6: the last event observed at Taybeh is correlated to the 1834 AD event:
The last event observed in Taybeh is dated during the 18th century AD, as no major earthquake is described in the southern Dead Sea area we proposed that it could be a minor event, like a Mw 5-6 earthquake, locally recorded. But Agnon 2014[26] proposed that the 1834 AD event correspond to the last event recorded in Tilah. In that case the last event at Taybeh might also correspond to the 1834 AD event. In figure S11 we present the corresponding slip deficit. The deficit of the northern Wadi Araba segment is reduced to 0.7m, which appears sensible. Nevertheless it increased noticeably the deficit in the Jordan Valley, where we have previously located the 1834 AD event. This scenario tends to increase the heterogeneity in the slip deficit along the fault. Moreover this location for the 1834 AD event is not fully consistent with the historical reports.

Alternative 7: impact of the occurrence of the 114 AD event:
Finally as explained previously the event E5 remains ambiguous because of the few cracks visible in the trench and the controversial historical descriptions. Thus we propose an alternative scenario, which does not take into account the 114 AD event, which is presented in figure S12. In this case the balance is realized on more than 2000 years, and the accumulated slip deficit increases noticeably, which might be another evidence of the occurrence of this earthquake. Nevertheless if we consider that the 363 AD event ruptured also the central part of the Wadi Araba, which is a plausible alternative as explained before, the effect of the 114 AD event is not visible anymore in our balance, as this one is realized for the period since the 4th century AD crisis.

These alternative models show that scenarios, which do not include large events, are associated to very large deficit, until 7m (Fig S8). These values appear quite unrealistic, which suggests that large earthquake occur episodically in the area.

6. Probability computation

To compute the probability of occurrence of an earthquake we followed the "empirical" model of Savage[53]. Savage proposed that the conditional probability of a future event might be estimated from the observed recurrence intervals. His method was later extended to include dating uncertainties of paleoseismic series[54]. The catalogue

presents a number n of intervals for each segment. We count the number m of n intervals that are shorter than the period since the last event (T, T+ΔT). With success defined as an event falling in the interval (T, T+ΔT), and failure otherwise, the distribution governing the probability p of a future event in (T, T+ΔT) follows a beta distribution[54]:

$$P(p|m,n) = \frac{(n+1)!}{m!(n-m)!} p^m (1-p)^{n-m}$$

For this distribution the mean and variance are respectively, $\bar{p} = (m+n)(n+1)$ and $\sigma^2 = \bar{p}(1-\bar{p})/(n-3)$. We define a confidence interval (C.I.) and determine p_{lo} and p_{hi}, as the 5% and 95% boundaries of P:

$$\int_{-\infty}^{plo} P(p|m,n)\mathrm{dp} = 0.05 \text{ and } \int_{-\infty}^{phi} P(p|m,n)\mathrm{dp} = 0.95$$

And C.I. corresponds to the integral of P(p|m,n) between p_{lo} and p_{hi}. We discuss only values of the C.I. that we consider as more representative to assess the seismic hazard. Usually the estimated probability improves with a longer record, but the large uncertainties in the empirical distribution of conditional probabilities show that even the present long paleoseismic series do not necessarily constrain uncertainties better when relatively few interval lengths are longer than the open interval since the last event.

7. References

1. Migowski, C., Stein, M., Prasad, S., Negendank, J. F. W. & Agnon, A. Holocene Climate Variability and Cultural Evolution in the Near East from the Dead Sea Sedimentary Record. *Quaternary Research* **66,** 421–431 (2006).
2. McCalpin, J. P. *Paleoseismology*. **95,** (Academic press, 2009).
3. Le Béon, M. *et al.* Slip rate and locking depth from GPS profiles across the southern Dead Sea Transform. *J. Geophys. Res.* **113,** 281–19 (2008).
4. Masson, F., Hamiel, Y., Agnon, A., Klinger, Y. & Deprez, A. Variable behavior of the Dead Sea Fault along the southern Arava segment from GPS measurements. *comptes rendus geoscience* **347,** 1–9 (2015).
5. Wells, D. L. & Coppersmith, K. J. New empirical relationships among magnitude, rupture length, rupture width, rupture area, and surface displacement. *Bulletin of the Seismological Society of America* **84,** 974–1002 (1994).
6. Ramsey, C. B. Bayesian Analysis of Radiocarbon Dates. *Radiocarbon* **51,** 337–360 (2009).
7. Kagan, E., Stein, M., Agnon, A. & Neumann, F. Intrabasin paleoearthquake and quiescence correlation of the late Holocene Dead Sea. *J. Geophys. Res.* **116,** 195–27 (2011).

8. Ben-Menahem, A. Four thousand years of seismicity along the Dead Sea Rift. *Journal of Geophysical Research: Solid Earth* **96,** 20195–20216 (1991).
9. Ambrasseys, N. *Earthquakes in the eastern Mediterranean and the Middle East: a multidisciplinary study of 2000 years of seismicity*. (2009).
10. Migowski, C., Agnon, A., Bookman, R., Negendank, J. F. W. & Stein, M. Recurrence pattern of Holocene earthquakes along the Dead Sea transform revealed by varve-counting and radiocarbon dating of lacustrine sediments. *Earth and Planetary Science Letters* **222,** 301–314 (2004).
11. Guidoboni, E., Comastri, A. & Traina, G. *Catalogue of ancient earthquakes in the Mediterranean area up to the 10th century*. (Istituto Nazionale di Geofisica, 1994).
12. Ferry, M., Meghraoui, M., Karaki, N. A., Al-Taj, M. & Khalil, L. Episodic behavior of the Jordan Valley section of the Dead Sea fault inferred from a 14-ka-long integrated catalog of large earthquakes. *Bulletin of the Seismological Society of America* **101,** 39–67 (2011).
13. Shaked, Y. *et al.* Large earthquakes kill coral reefs at the north-west Gulf of Aqaba. *Terra Nova* **16,** 133–138 (2004).
14. Shaked, Y., Lazar, B., Marco, S., Stein, M. & Agnon, A. Late Holocene events that shaped the shoreline at the northern Gulf of Aqaba recorded by a buried fossil reef. *Israel Journal of Earth Sciences* **58,** 355–368 (2011).
15. Klinger, Y., Le Beon, M. & Al-Qaryouti, M. 5000 yr of paleoseismicity along the southern Dead Sea fault. *Geophys. J. Int.* **202,** 313–327 (2015).
16. Russell, K. W. The earthquake chronology of Palestine and northwest Arabia from the 2nd through the mid-8th century AD. *bulletin of the American schools of oriental research* **260,** 37–59 (1985).
17. Yadin, Y. The Excavation of Masada—1963/64: Preliminary Report. *Israel Exploration Journal* **15,** 1–120 (1965).
18. Thomas, R., Niemi, T. M. & Parker, S. T. Structural damage from earthquakes in the second: ninth centuries at the archaeological site of Aila in Aqaba, Jordan. *bulletin of the American schools of oriental research* **346,** 59–77 (2007).
19. Amiran, D. H., Arieh, E. & Turcotte, T. Earthquakes in Israel and adjacent areas: Macroseismic observations since 100 BCE. *Israel Exploration Journal* 260–305 (1994).
20. Langgut, D., Yannai, E., Taxel, I., Agnon, A. & Marco, S. Resolving a historical earthquake date at Tel Yavneh (central Israel) using pollen seasonality. *Palynology* **40,** 145–159 (2015).
21. Wechsler, N., Rockwell, T. K. & Klinger, Y. Variable slip-rate and slip-per-event on a plate boundary fault: The Dead Sea fault in northern Israel. *Tectonophysics* **722,** 210–226 (2018).
22. Haynes, J. M., Niemi, T. M. & Atallah, M. Evidence for ground-rupturing earthquakes on the Northern Wadi Araba fault at the archaeological site of Qasr Tilah, Dead Sea Transform fault system, Jordan. *J Seismol* **10,** 415–430 (2006).
23. Marco, S., Rockwell, T., Heimann, A., Frieslander, U. & Agnon, A. Late Holocene activity of the Dead Sea Transform revealed in 3D palaeoseismic trenches on the Jordan Gorge segment. *Earth and Planetary Science Letters* **234,** 189–205 (2005).
24. Marco, S. Recognition of earthquake-related damage in archaeological sites: Examples from the Dead Sea fault zone. *Tectonophysics* **453,** 148–156 (2008).
25. Wechsler, N. *et al.* A Paleoseismic Record of Earthquakes for the Dead Sea Transform Fault between the First and Seventh Centuries C.E.: Nonperiodic Behavior of a Plate Boundary Fault. *Bulletin of the Seismological Society of America*

104, 1329–1347 (2014).
26. Agnon, A. in *Dead Sea Transform Fault System: Reviews* (eds. Garfunkel, Z., Ben Avraham, Z. & Kagan, E.) **6,** 207–261 (Springer Netherlands, 2014).
27. Guidoboni, E. & Comastri, A. *Catalogue of Earthquakes and Tsunamis in the Mediterranean Area from the 11th to the 15th Century*. (Istituto nazionale di geofisica e vulcanologia, 2005).
28. Amit, R., Zilberman, E. & Enzel, Y. Paleoseismic evidence for time dependency of seismic response on a fault system in the southern Arava Valley, Dead Sea rift, Israel. *Geological Society of America Bulletin* **114,** 192–206 (2002).
29. Zilberman, E., Amit, R., Porat, N., Enzel, Y. & Avner, U. Surface ruptures induced by the devastating 1068 AD earthquake in the southern Arava valley, Dead Sea Rift, Israel. *Tectonophysics* **408,** 79–99 (2005).
30. Klinger, Y., Avouac, J. P., Dorbath, L., Abou Karaki, N. & Tisnerat, N. Seismic behaviour of the Dead Sea fault along Araba valley, Jordan. *Geophys. J. Int.* **142,** 769–782 (2000).
31. Ambrasseys, N. *Earthquakes in the eastern Mediterranean and the Middle East: a multidisciplinary study of 2000 years of seismicity*. (2009).
32. Kanari, M., Ben-Avraham, Z., Tibor, G. & Bookman, R. On-land & Offshore Evidence for Holocene Earthquakes in the Northern Gulf of Aqaba-Elat, Israel/Jordan. in (2015).
33. Zeng, J. L., Shao, Y., Klinger, Y. & Xie, K. Variability in magnitude of paleoearthquakes revealed by trenching and historical records, along the Haiyuan fault, China. *Journal of Geophysical Research: Solid Earth* **120,** (2015).
34. Karcz, I. Implications of some early Jewish sources for estimates of earthquake hazardin the Holy Land. *Annals of Geophysics* **47,** 759–792 (2004).
35. Sbeinati, M. R., Darawcheh, R. & Mouty, M. The historical earthquakes of Syria: an analysis of large and moderate earthquakes from 1365 BC to 1900 AD. *Annals of Geophysics* **48,** (2005).
36. Marco, S. & Klinger, Y. in *Dead Sea Transform Fault System: Reviews* (eds. Garfunkel, Z., Ben Avraham, Z. & Kagan, E.) **6,** 183–205 (Springer Netherlands, 2014).
37. Zohar, M., Salamon, A. & Rubin, R. Reappraised list of historical earthquakes that affected Israel and its close surroundings. *J Seismol* **20,** 971–985 (2016).
38. Ben Avraham, Z., Brink, ten, U., Bell, R. & Reznikov, M. Gravity field over the Sea of Galilee: Evidence for a composite basin along a transform fault. *Journal of Geophysical Research: Solid Earth* **101,** 533–544 (1996).
39. Avni, R., Bowman, D., Shapira, A. & Nur, A. Erroneous interpretation of historical documents related to the epicenter of the 1927 Jericho earthquake in the Holy Land. *J Seismol* **6,** 469–476 (2002).
40. Zohar, M. & Marco, S. Re-estimating the epicenter of the 1927 Jericho earthquake using spatial distribution of intensity data. *Journal of Applied Geophysics* **82,** 19–29 (2012).
41. Abou Karaki, N. Synthèse et carte sismotectonique des pays de la bordure orientale de la Méditerranée: sismicité du système de failles du Jourdain-Mer Morte. (Strasbourg 1, 1987).
42. Marco, S., Agnon, A., Ellenblum, R. & Eidelman, A. 817-year-old walls offset sinistrally 2.1 m by the Dead Sea Transform, Israel. *Journal of Geodynamics* **24,** 11–20 (1997).
43. Ellenblum, R., Marco, S., Agnon, A. & Rockwell, T. Crusader castle torn apart by

earthquake at dawn, 20 May 1202. *Geology* **26,** 303–306 (1998).
44. Daeron, M. *et al.* Sources of the large A.D. 1202 and 1759 Near East earthquakes. *Geology* **33,** 529–8 (2005).
45. Nemer, T., Meghraoui, M. & Khair, K. The Rachaya-Serghaya fault system (Lebanon): Evidence of coseismic ruptures, and the AD 1759 earthquake sequence. *J. Geophys. Res.* **113,** 61–12 (2008).
46. Gomez, F. *et al.* Coseismic displacements along the Serghaya Fault: an active branch of the Dead Sea Fault System in Syria and Lebanon. *Journal of the Geological Society* **158,** 405–408 (2001).
47. Gomez, F., Meghraoui, M. & Darkal, A. N. Holocene faulting and earthquake recurrence along the Serghaya branch of the Dead Sea fault system in Syria and Lebanon. *Geophys. J. Int.* **153,** 658–674 (2003).
48. Daeron, M. *et al.* 12,000-Year-Long Record of 10 to 13 Paleoearthquakes on the Yammouneh Fault, Levant Fault System, Lebanon. *Bulletin of the Seismological Society of America* **97,** 749–771 (2007).
49. Elias, A. *et al.* Active thrusting offshore Mount Lebanon: Source of the tsunamigenic A.D. 551 Beirut-Tripoli earthquake. *Geology* **35,** 755–4 (2007).
50. Ellenblum, R. *et al.* Archaeological record of earthquake ruptures in Tell Ateret, the Dead Sea Fault. *Tectonics* **34,** 2105–2117 (2015).
51. Nemer, T. & Meghraoui, M. Evidence of coseismic ruptures along the Roum fault (Lebanon): a possible source for the AD 1837 earthquake. *Journal of Structural Geology* **28,** 1483–1495 (2006).
52. Biasi, G. P. & Wesnousky, S. G. Steps and Gaps in Ground Ruptures: Empirical Bounds on Rupture Propagation. *Bulletin of the Seismological Society of America* **106,** 1110–1124 (2016).
53. Savage, J. C. Empirical earthquake probabilities from observed recurrence intervals. *Bulletin of the Seismological Society of America* **84,** 219–221 (1994).
54. Biasi, G. P., Weldon, R. J. & Fumal, T. E. Paleoseismic event dating and the conditional probability of large earthquakes on the southern San Andreas fault, California. *Bulletin of the Seismological Society of America* **92,** 2761–2781 (2002).
55. Reimer, P. J. *et al.* IntCal13 and Marine13 Radiocarbon Age Calibration Curves 0–50,000 Years cal BP. *Radiocarbon* **55,** 1869–1887 (2013).
56. Stuiver, M. & Polach, H. A. Discussion Reporting of 14C data. *Radiocarbon* **19,** 355–363 (1977).

8. Table and figures

Sample name	Laboratory Code	Fraction Modern	±	$\delta^{14}C$ (‰)	±	^{14}C age (BP)	±	modeled calibrated age(±2σ)
TAY14-02	UCIAMS 164533	0.8592	0.0012	-140.8	1.2	1220	15	718 - 878
TAY14-11	UCIAMS 164534	0.7652	0.0011	-234.8	1.1	2150	15	145 BC - 61 BC
TAY14-9	UCIAMS 158597	0.9486	0.0017	-51.4	1.7	425	15	1436 - 1475
TAY14-17	UCIAMS 164535	0.4071	0.0058	-592.9	5.8	7220	120	6256 BC - 5759 BC
TAY14-30	UCIAMS 158598	0.613	0.0009	-387	0.9	3930	15	2477 BC - 2347 BC
TAY14-37	UCIAMS 158599	0.6899	0.0021	-310.1	2.1	2980	25	1277 BC - 1121 BC*
TAY14-38	UCIAMS 158600	0.7587	0.001	-241.3	1,0	2220	15	362 BC - 205 BC
TAY14-41	UCIAMS 158601	0.5208	0.004	-479.2	4,0	5240	70	4247 BC - 3820 BC
TAY14-42	UCIAMS 158602	0.366	0.0008	-634	0.8	8075	20	7079 BC - 7041 BC
TAY14-43	UCIAMS 158603	0.4878	0.0008	-512.2	0.8	5765	15	4685 BC - 4552 BC
TAY14-44	UCIAMS 158604	0.9328	0.0013	-67.2	1.3	560	15	1319 - 1415
TAY14-51	UCIAMS 158605	0.4478	0.0008	-552.2	0.8	6455	15	5476 BC - 5376 BC
TAY14-54	UCIAMS 158606	0.7799	0.0011	-220.1	1.1	1995	15	164 BC - 81 BC
TAY14-55	UCIAMS 158607	0.7933	0.0011	-206.7	1.1	1860	15	90 - 228
TAY14-56	UCIAMS 158608	0.9741	0.0017	-25.9	1.7	210	15	1765 - 1953
TAY14-58	UCIAMS 158609	0.7756	0.0011	-224.4	1.1	2040	15	100 BC - 4 AD
TAY14-59	UCIAMS 158610	0.7293	0.0012	-270.7	1.2	2535	15	910 BC - 833 BC
TAY14-60	UCIAMS 158611	0.6277	0.001	-372.3	1,0	3740	15	2202 BC - 2050 BC*
TAY14-61	UCIAMS 158612	0.9777	0.0019	-22.3	1.9	180	20	1774 - 1953
TAY14-63	UCIAMS 158613	0.7541	0.0011	-245.9	1.1	2270	15	381 BC - 232 BC
TAY14-65	UCIAMS 158614	0.7385	0.0011	-261.5	1.1	2435	15	731 BC - 413 BC
TAY14-66	UCIAMS 158615	0.7562	0.0011	-243.8	1.1	2245	15	391 BC - 258 BC
TAY14-69	UCIAMS 158616	0.9824	0.0014	-17.6	1.4	145	15	1666 - 1809
TAY14-70	UCIAMS 164536	0.9825	0.0014	-17.5	1.4	140	15	1677 - 1875
TAY14-71	UCIAMS 164537	0.7583	0.0011	-241.7	1.1	2220	15	365 BC - 206 BC*
TAY14-73	UCIAMS 158617	0.742	0.0029	-258	2.9	2400	35	741 BC - 398 BC
TAY14-74	UCIAMS 158618	0.7683	0.0012	-231.7	1.2	2115	15	147 BC - 56 BC
TAY14-75	UCIAMS 158619	0.7074	0.001	-292.6	1,0	2780	15	994 BC - 894 BC
TAY14-79	UCIAMS 164538	0.8618	0.0013	-138.2	1.3	1195	15	774 - 881*
TAY14-100	UCIAMS 158620	0.3684	0.0007	-631.6	0.7	8020	20	7058 BC - 6830 BC
TAY14-119	UCIAMS 164539	0.8045	0.0014	-195.5	1.4	1750	15	242 - 337
TAY14-123	UCIAMS 158621	0.7592	0.0012	-240.8	1.2	2215	15	361 BC - 258 BC

Table S1 : Mass Spectroscopy measurements were made at the Keck carbon cycle mass spectroscopy facility at University of California, Irvine. Ages were calibrated using OxCal 4.2[6] and calibration curve INTCAL13[55]. Calibrated ages are given in date Before Christ (BC) or After Christ (AD). Radiocarbon concentrations are given as fractions of the

Modern standard, D14C, and conventional radiocarbon age, following the conventions of Stuiver and Polach[56]. Sample preparation backgrounds have been subtracted, based on measurements of ^{14}C-free wood. All results have been corrected for isotopic fractionation according to the conventions of Stuiver and Polach[56], with $\delta^{13}C$ values measured on prepared graphite using the AMS spectrometer. These can differ from $\delta^{13}C$ of the original material, if fractionation occurred during sample graphitization or the AMS measurement, and are not shown.

* Samples that are out of stratigraphic order, most probably reworked. They are not included in the age model.

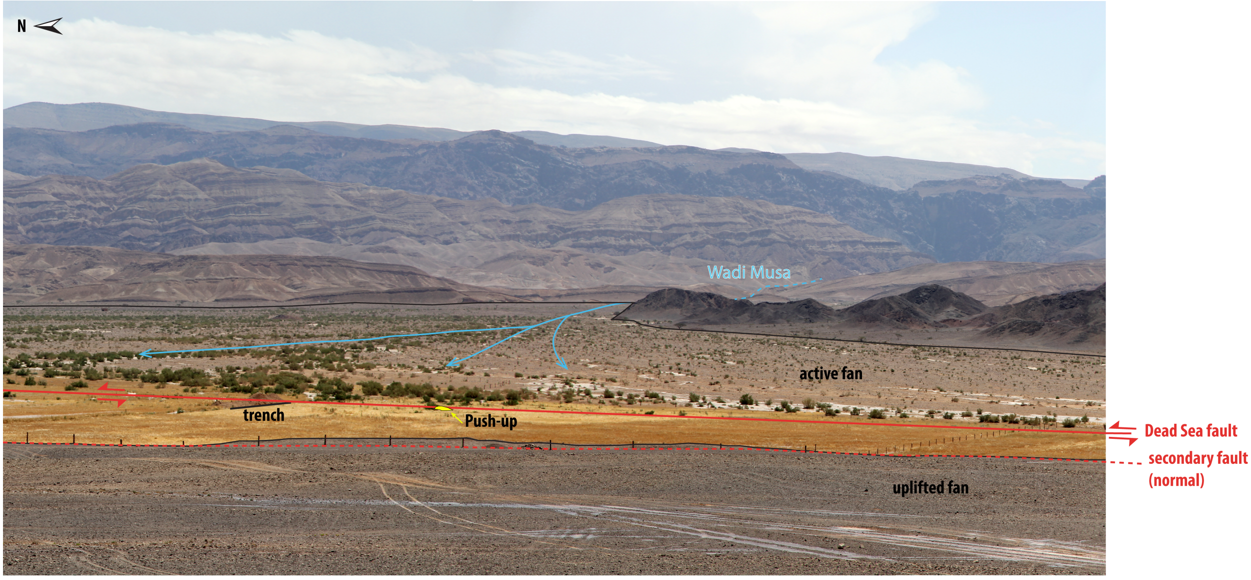

Figure S1: Field view of the alluvial fan of the Wadi Musa. The picture is taken from the uplifted fan, the trenching place is visible on the active fan, as well as a small pressure ridge (~60cm high).

Figure S2: Photomosaic of the trench southern wall (top) and mapping of stratigraphic layers, faults, and earthquakes (bottom). The event horizons associated to the seismic events recorded in the trench are indicated in red on the stratigraphic log.

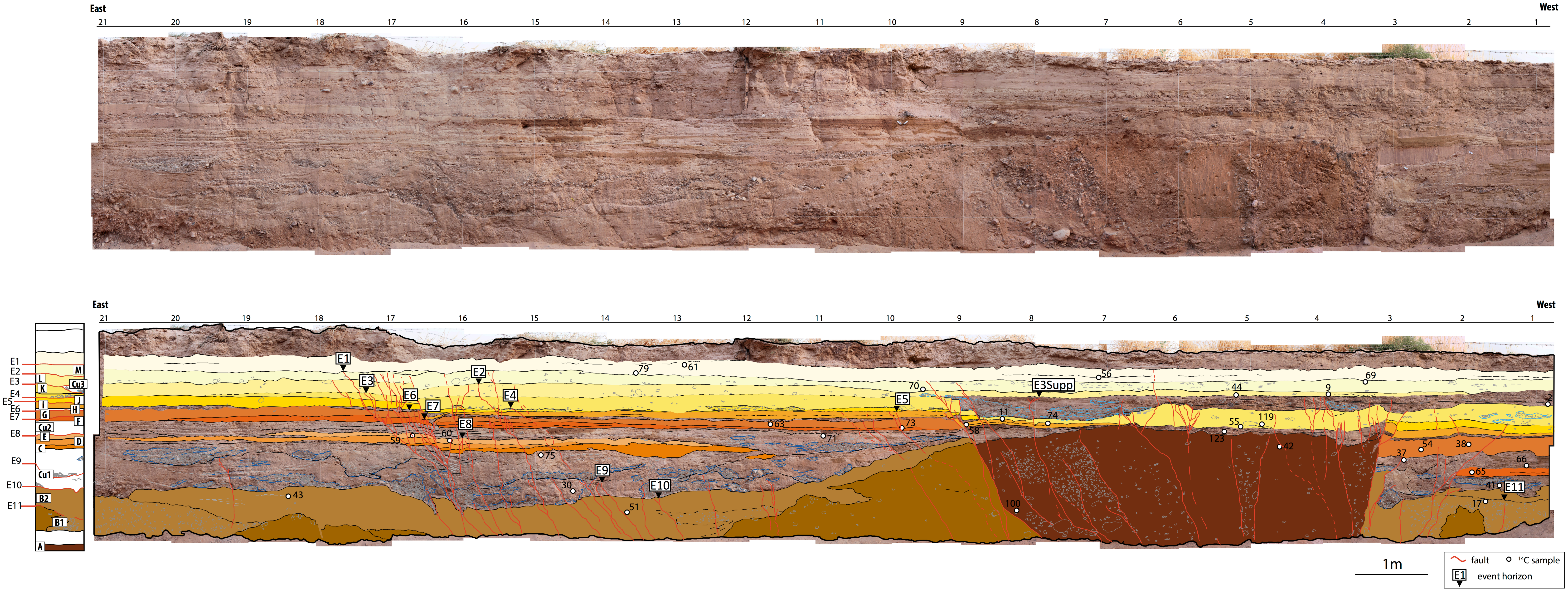
East
West
21 20 19 18 17 16 15 14 13 12 11 10 9 8 7 6 5 4 3 2 1
E1
E2
E3
E4
E5
E6
E7
E8
E9
E10
E11
E3Supp
A B1 B2 Cu1 C D E Cu2 F G H i J K Cu3 L M
1m
fault
¹⁴C sample
event horizon

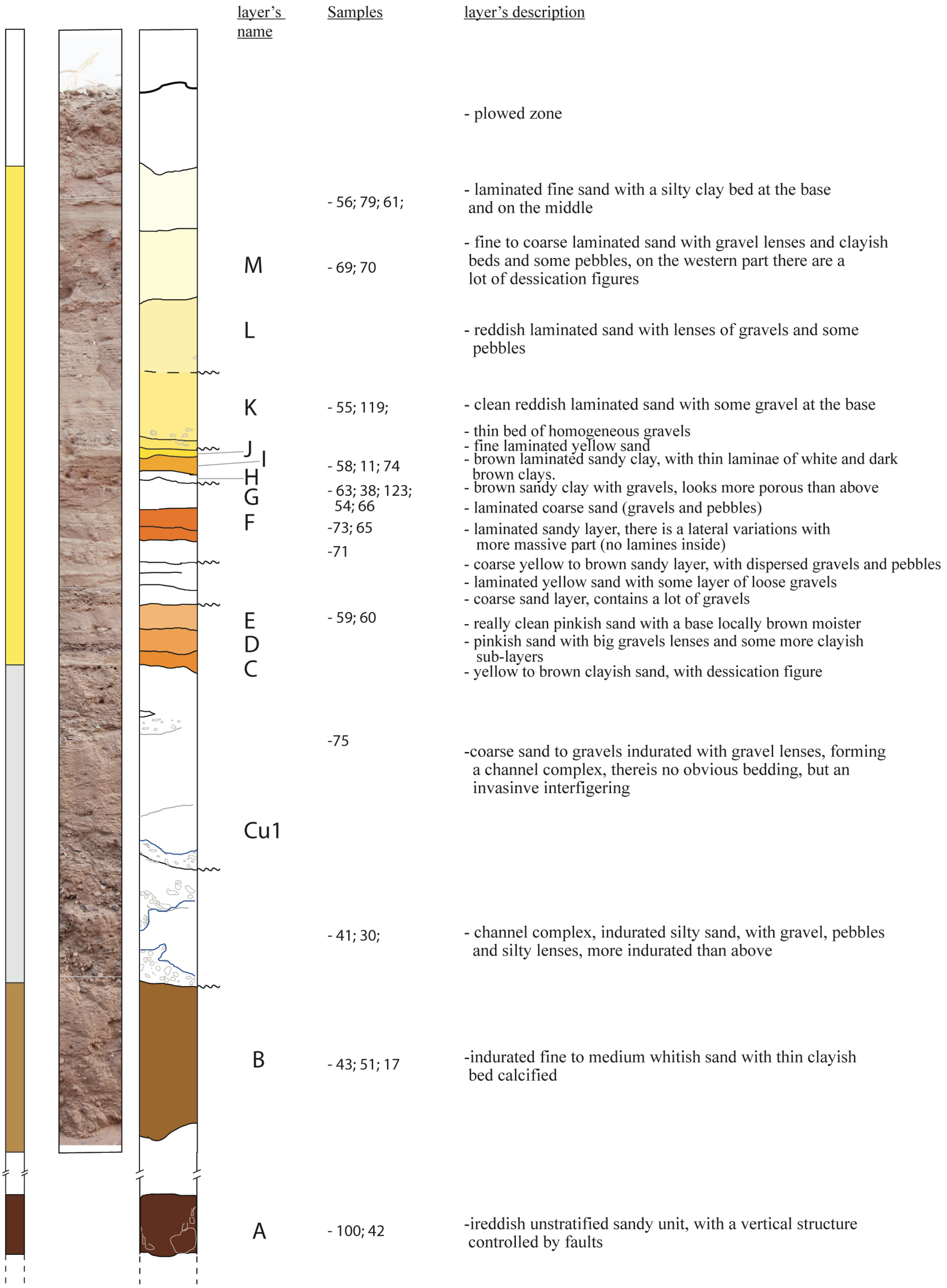

Figure S3: Stratigraphic log of the trench, on the left simplified log with the limits of the four main units, and then the complete log is represented. Letters A to M names the different stratigraphic layers. The layers are quite continuous laterally along the trench, the ^{14}C samples dated in each layer are indicated.

Figure S4: Age model computed for the trench stratigraphy using OxCal v4.2[6] and IntCal13 calibration curve[54]. Dark grey indicates modeled ages including stratigraphic information and red are the modeled age for seismic events. Phases indicate subsets of samples where no stratigraphic order is imposed.

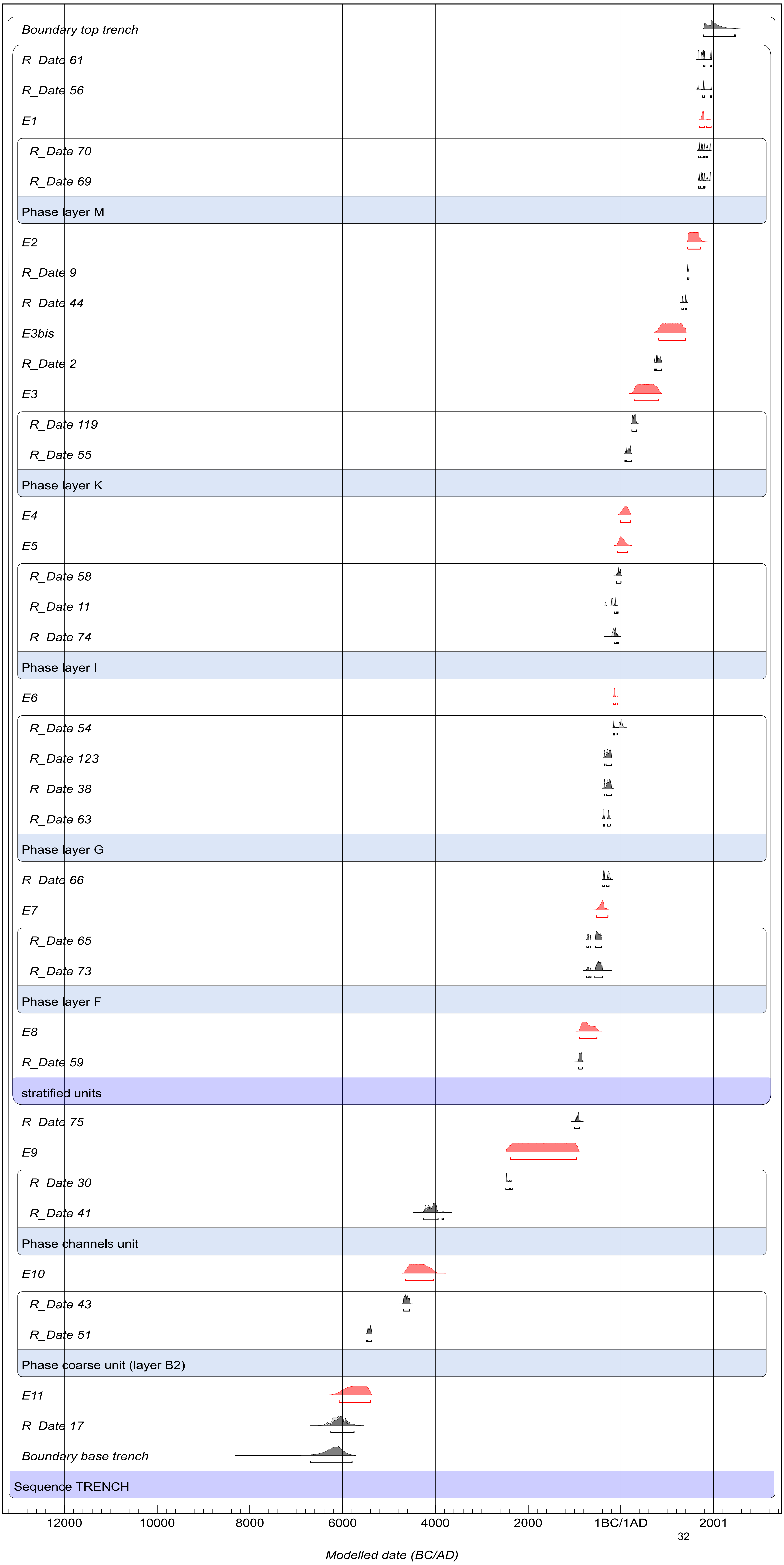
Boundary top trench
R_Date 61
R_Date 56
E1
R_Date 70
R_Date 69
Phase layer M
E2
R_Date 9
R_Date 44
E3bis
R_Date 2
E3
R_Date 119
R_Date 55
Phase layer K
E4
E5
R_Date 58
R_Date 11
R_Date 74
Phase layer I
E6
R_Date 54
R_Date 123
R_Date 38
R_Date 63
Phase layer G
R_Date 66
E7
R_Date 65
R_Date 73
Phase layer F
E8
R_Date 59
stratified units
R_Date 75
E9
R_Date 30
R_Date 41
Phase channels unit
E10
R_Date 43
R_Date 51
Phase coarse unit (layer B2)
E11
R_Date 17
Boundary base trench
Sequence TRENCH
12000
10000
8000
6000
4000
2000
1BC/1AD
2001
Modelled date (BC/AD)

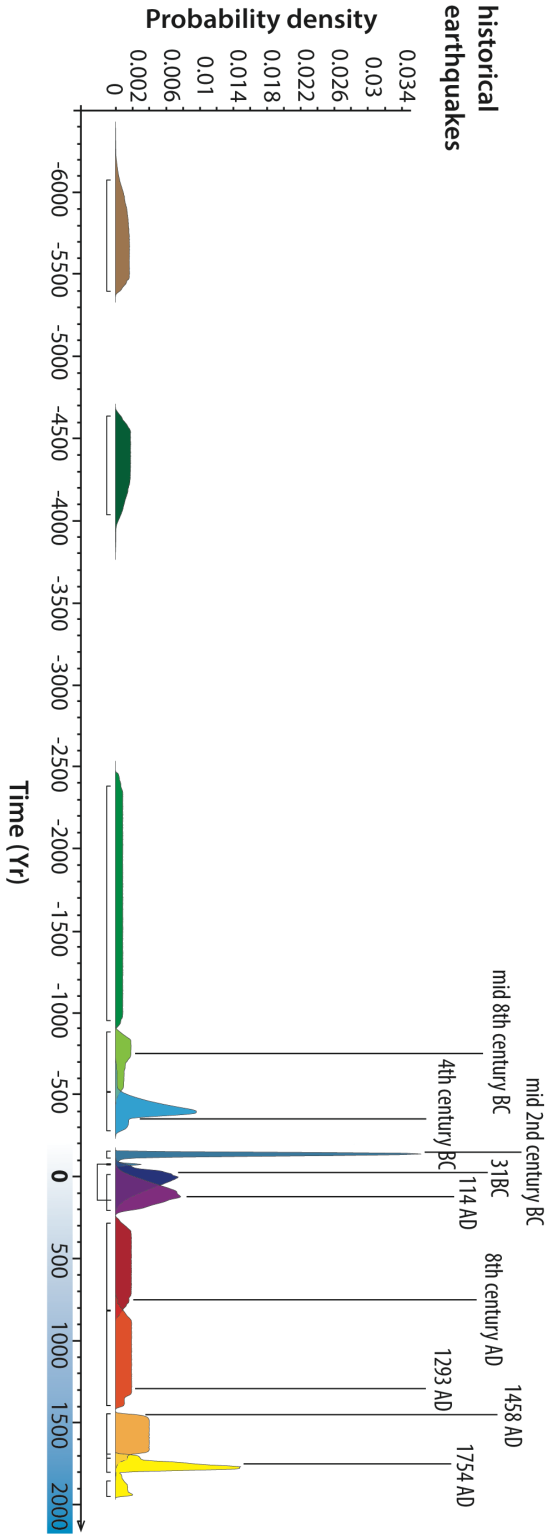

Figure S5: Computed age model from OxCal v4.2[6] for the seismic events recorded in the trench. The period highlighted in blue corresponds to the times for which we expect that the historical earthquakes record is relatively complete.

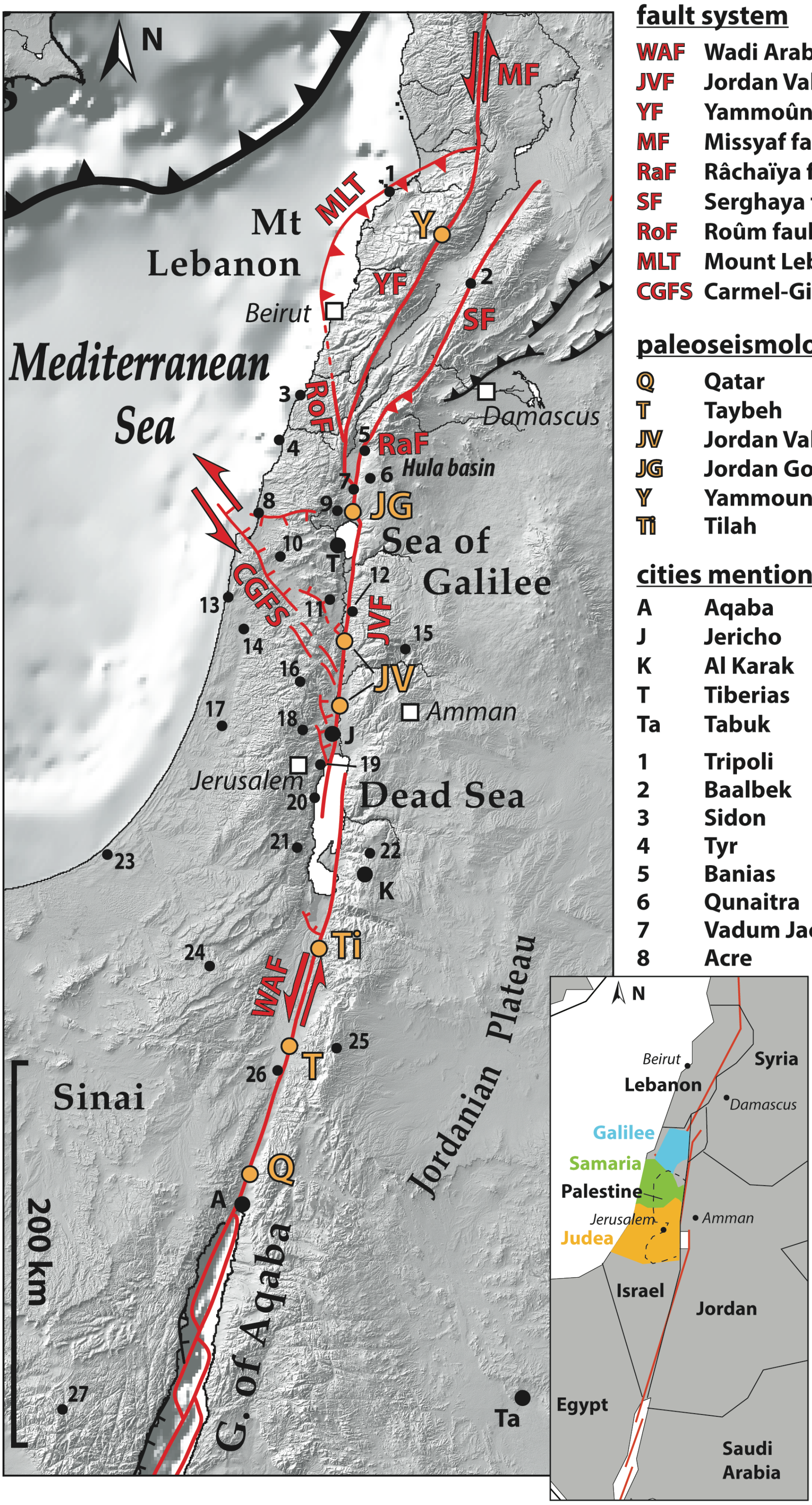

Figure S6: Topographic map of the studied area from SRTM3, faults geometries are derived from Klinger et al.[15]. The locations mentioned in the text are reported on the map. The inset map presents the geographical areas mentioned in the text. Figure was generated with Adobe illustrator CS6 (http://www.adobe.com/fr/products/illustrator.html)

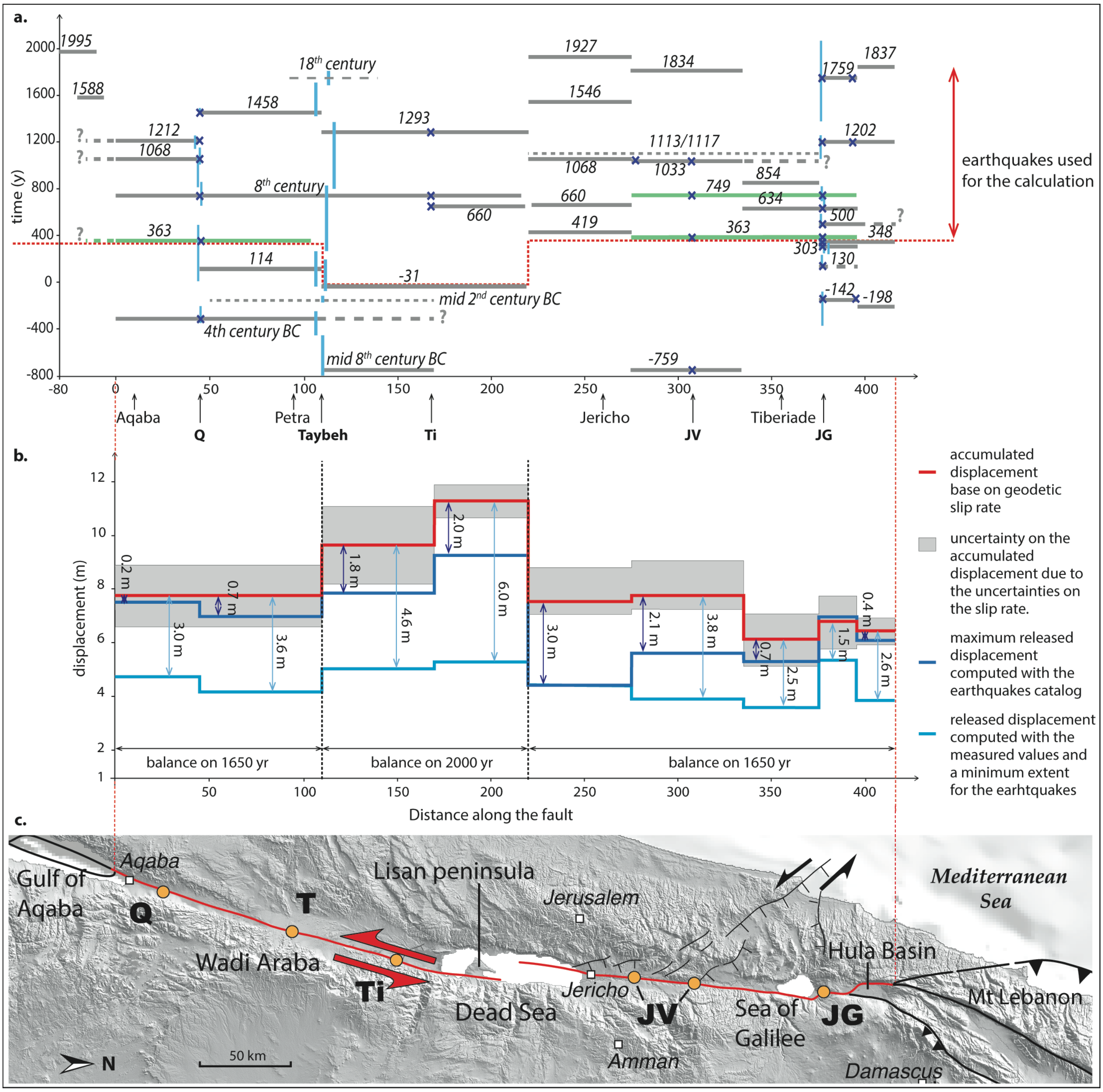

Figure S7: alternative earthquake catalogue for the Dead Sea fault, we consider a longer extent for the two 363 AD events and the 749 AD event. **a.** Historical earthquakes distribution in space and time, each horizontal bar corresponds to an approximate earthquake location along the Dead Sea fault, dashed lines are used for uncertain locations or lateral limits. The vertical bars correspond to the time interval associated to the paleoearthquakes at Taybeh, obtained after a Bayesian modeling in Oxcal. For Qatar the time intervals are from Klinger et al.[15], for the Jordan Gorge the ages are from Marco et al.[23] and from Wechsler et al.[21]. The blue crosses represent the earthquakes visible in the different trenches. **b.** Accumulated displacement (red) from tectonic loading and accumulated released slip (blue) due to major earthquakes from 350 AD to 2015 AD along DSF, between the Gulf of Aqaba and the Hula basin. For all earthquakes two

scenarios are considered, the dark blue line shows the released displacement linked to a maximum lateral extent, the light blue line shows the released displacement linked to a minimum lateral extent. The slip deficit is written in meter for all the sections and for the two scenarios. **c.** Simplified structural map of the southern Dead Sea fault, the studied branches are highlighted in red, the sites of previous paleoseismological studies in the area are shown, Q: Qatar, Ti: Tilah, JV: Jordan valley. The topography is from SRTM3 and the faults geometries are derived from Le Béon et al.[15]. Figure was generated with Adobe illustrator CS6 (http://www.adobe.com/fr/products/illustrator.html)

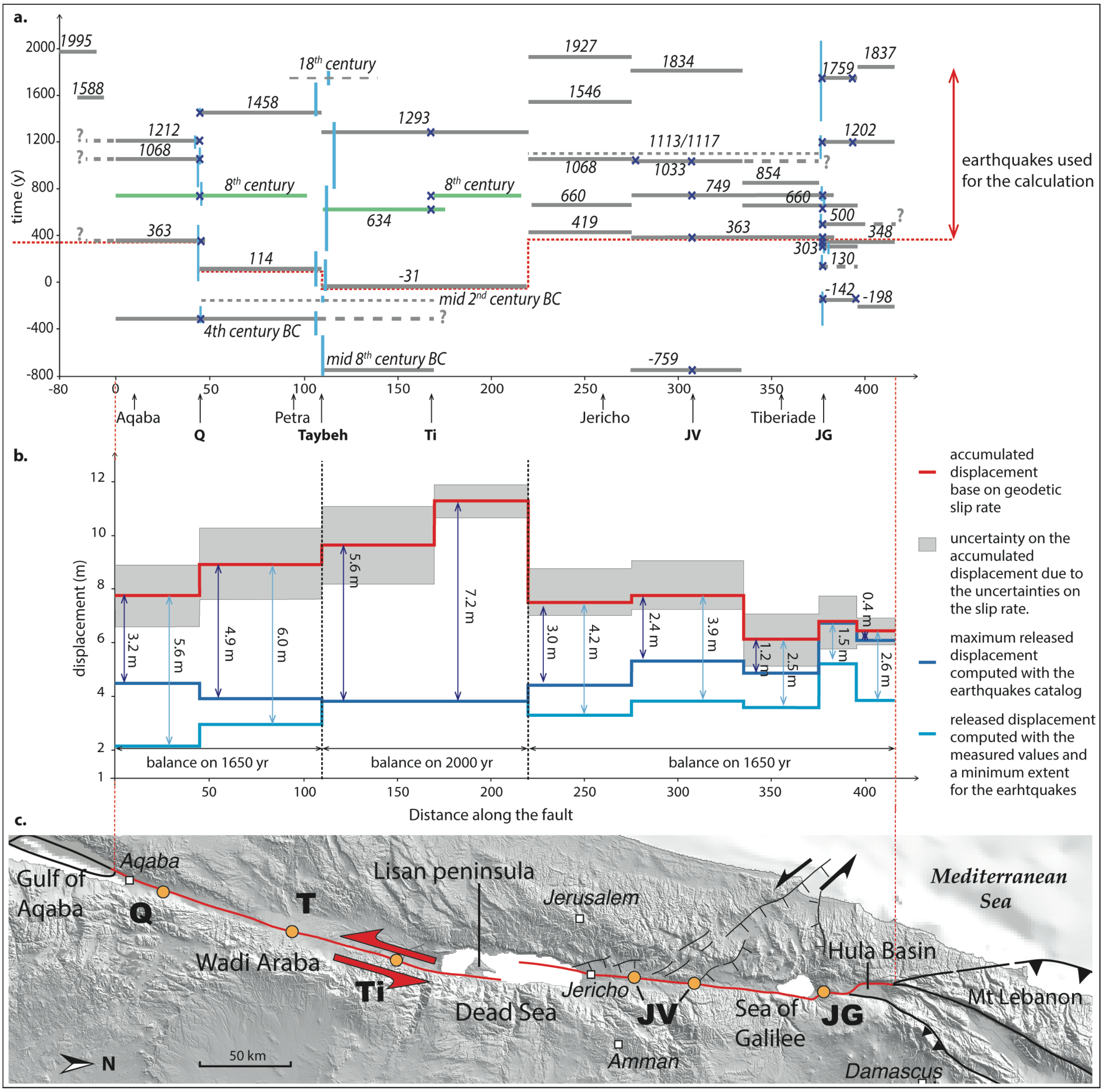

Figure S8: Same as figure S7; here the alternative scenario considers different locations for the 7th and 8th century AD events in the Wadi Araba.

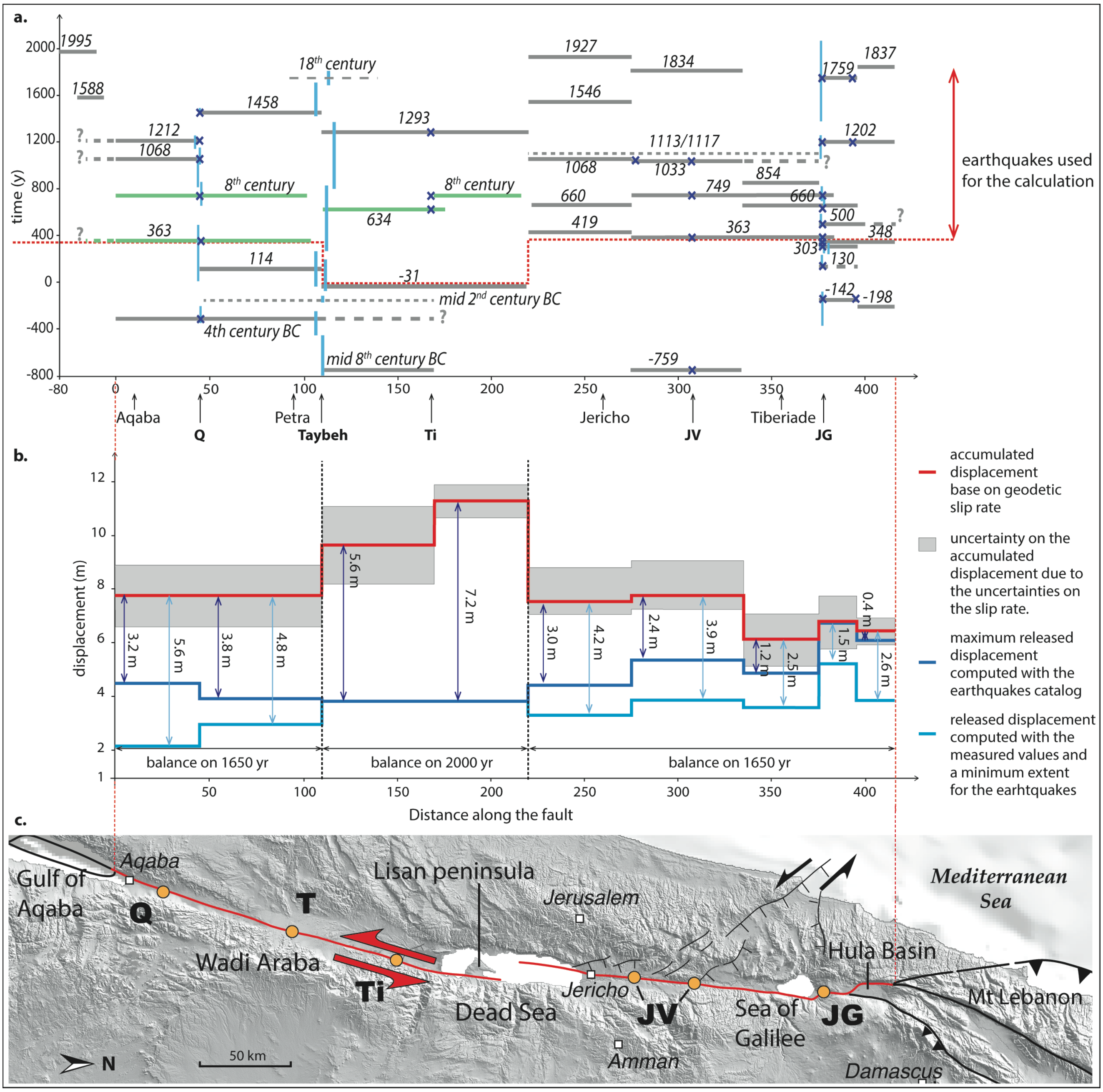

Figure S9: Same as figure S7; here the alternative scenario considers different locations for the 7th and 8th century AD earthquakes in the Wadi Araba, and a larger extension for the 363 AD earthquake.

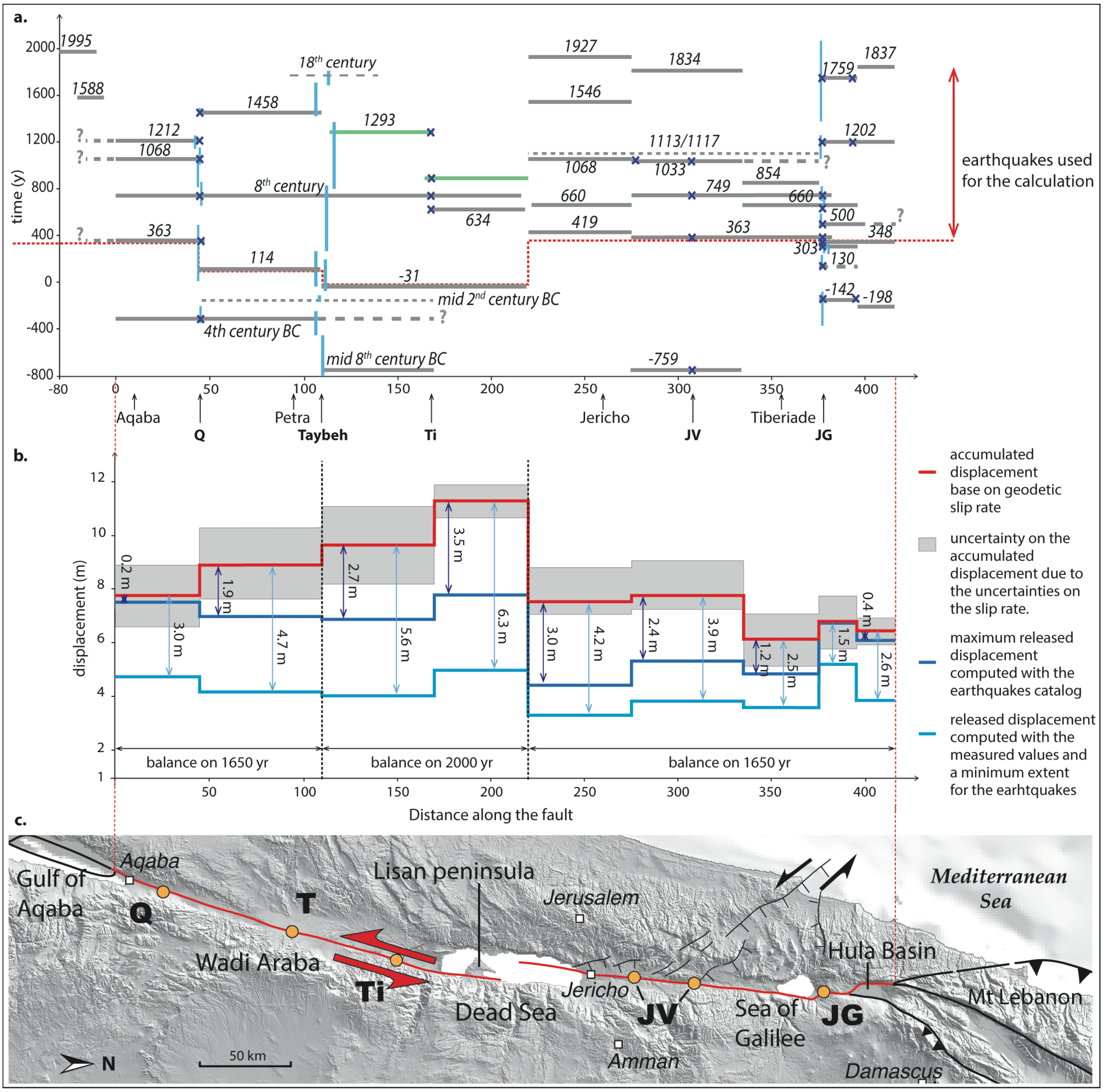

Figure S10: Same as figure S7; here the alternative scenario presents the effect of a smaller 1293 AD earthquake.

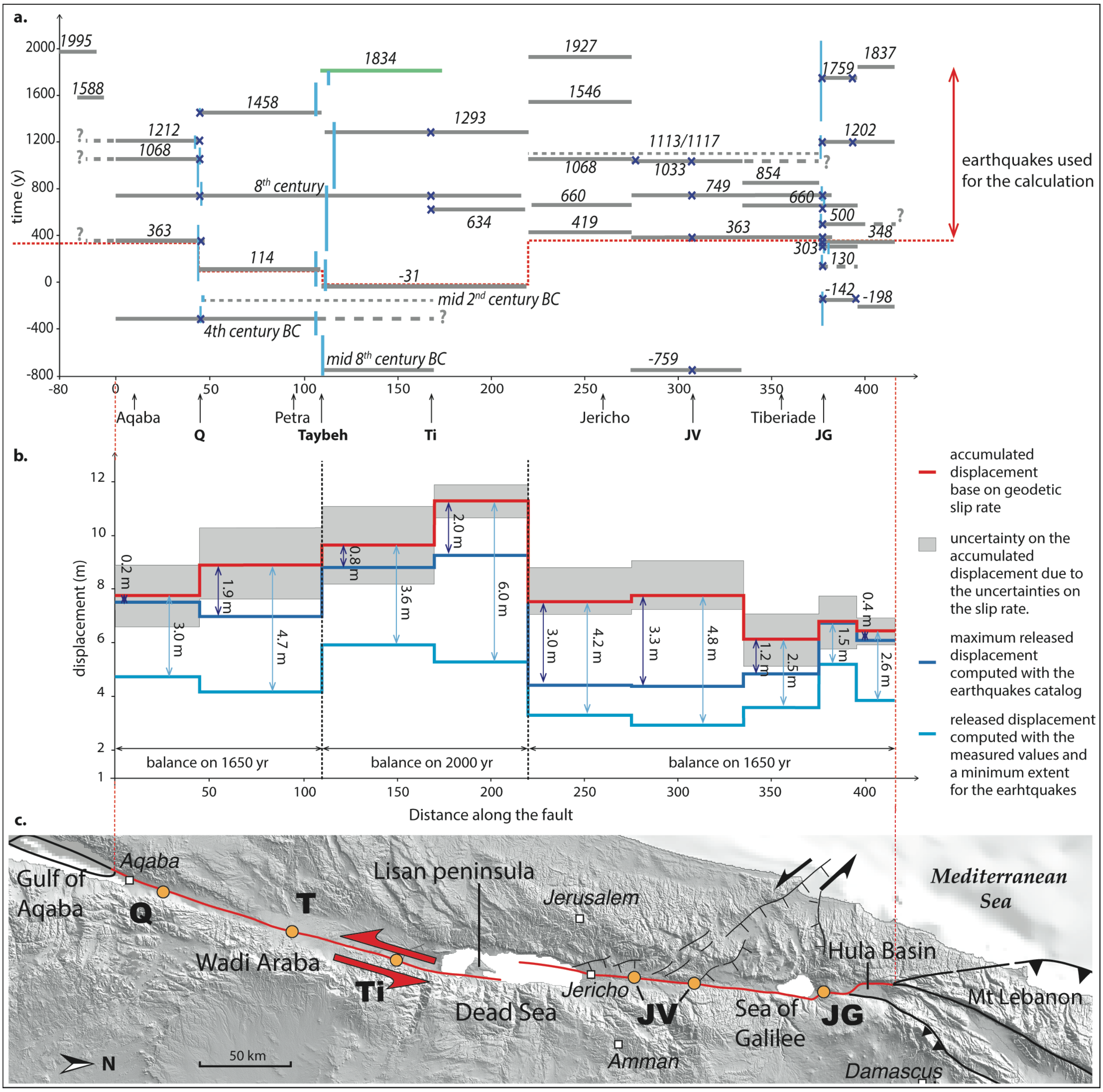

Figure S11: Same as figure S7; here the alternative scenario considers a different location for the1834 AD earthquake.

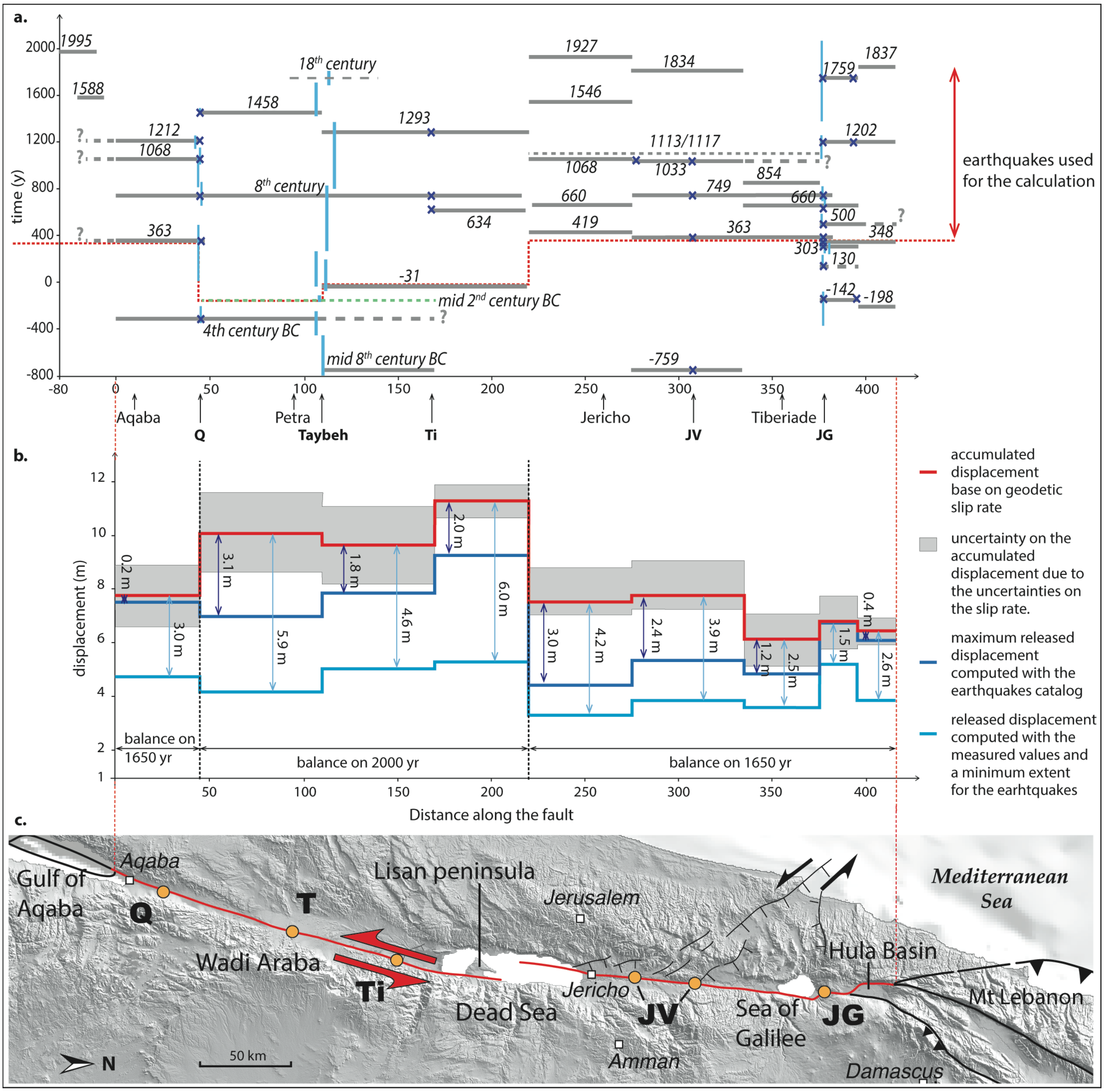

Figure S12: Same as figure S7; here we present an alternative scenario, which does not consider that E5 was an independent event, thus the catalogue does not contain the 114 AD earthquake.
